# *Paralemnalia thyrsoides*-associated fungi: phylogenetic diversity, cytotoxic potential, metabolomic profiling and docking analysis

**DOI:** 10.1186/s12866-023-03045-y

**Published:** 2023-10-26

**Authors:** Radwa Taher Mohie el-dien, Basma Khalaf Mahmoud, Miada F. Abdelwahab, Amgad I. M. Khedr, Mohamed Salah Kamel, Ramadan Yahia, Nada M. Mohamed, Amr El Zawily, Eman S. Kamel, Aliasger K Salem, Usama Ramadan Abdelmohsen, Mostafa A. Fouad

**Affiliations:** 1 Department of Pharmacognosy, Faculty of pharmacy, New Valley University, New Valley City, Egypt; 2 Department of Pharmacognosy, Faculty of Pharmacy, Minia University, 61519 Minia, Egypt; 3 Department of Pharmacognosy, Faculty of Pharmacy, Port Said University, 42526 Port Said, Egypt; 4 Department of Pharmacognosy, Faculty of Pharmacy, Deraya University, 61111 New Minia City, Minia Egypt; 5 Department of Microbiology and immunology, Faculty of Pharmacy, Deraya University, 61111 New Minia City, Minia Egypt; 6 Department of Pharmaceutical Chemistry, Faculty of Pharmacy, Modern University for Technology and Information (MTI), Cairo, Egypt; 7 Department of Plant and Microbiology, Faculty of Science, Damanhour University, 22511 Damanhour, Egypt; 8 Division of Pharmaceutics and Translational Therapeutics, College of Pharmacy, University of Iowa, Iowa City, IA 52242 USA

**Keywords:** Coral-associated fungi, *Paralemnalia thyrsoides*, Cytotoxicity, Metabolomics, Molecular docking

## Abstract

**Background:**

Cancer continues to be one of the biggest causes of death that affects human health. Chemical resistance is still a problem in conventional cancer treatments. Fortunately, numerous natural compounds originating from different microbes, including fungi, possess cytotoxic characteristics that are now well known. This study aims to investigate the anticancer prospects of five fungal strains that were cultivated and isolated from the Red Sea soft coral *Paralemnalia thyrsoides*. The in vitro cytotoxic potential of the ethyl acetate extracts of the different five isolates were evaluated using MTS assay against four cancer cell lines; A549, CT-26, MDA-MB-231, and U87. Metabolomics profiling of the different extracts using LC-HR-ESI-MS, besides molecular docking studies for the dereplicated compounds were performed to unveil the chemical profile and the cytotoxic mechanism of the soft coral associated fungi.

**Results:**

The five isolated fungal strains were identified as *Penicillium griseofulvum* (RD1), *Cladosporium sphaerospermum* (RD2), *Cladosporium liminiforme* (RD3), *Penicillium chrysogenum* (RD4), *and Epicoccum nigrum* (RD5). The in vitro study showed that the ethyl acetate extract of RD4 exhibited the strongest cytotoxic potency against three cancer cell lines A549, CT-26 and MDA-MB-231 with IC_50_ values of 1.45 ± 8.54, 1.58 ± 6.55 and 1.39 ± 2.0 µg/mL, respectively, also, RD3 revealed selective cytotoxic potency against A549 with IC_50_ value of 6.99 ± 3.47 µg/mL. Docking study of 32 compounds dereplicated from the metabolomics profiling demonstrated a promising binding conformation with EGFR tyrosine kinase that resembled its co-crystallized ligand albeit with better binding energy score.

**Conclusion:**

Our results highlight the importance of soft coral-associated fungi as a promising source for anticancer metabolites for future drug discovery.

**Supplementary Information:**

The online version contains supplementary material available at 10.1186/s12866-023-03045-y.

## Introduction

Cancer is defined as a group of disorders that can affect different body organs and are characterized by the invasion of healthy tissues and the unchecked proliferation of aberrant cells. New tumors can be formed as a result of cancer cells spreading to different bodily regions [1, 2]. With an estimated 10 million deaths from cancer in 2020, it is the most prevalent cause of death worldwide [3]. According to the World Cancer Report 2014, which has been issued by the International Agency for Research on Cancer of the World Health Organization, the number of new cancer cases worldwide increased to an estimated 14 million in 2012 and is predicted to reach 19.3 million cases annually by 2025 [4]. According to the same research, lung cancer accounted for 13% of all cases of cancer in 2012, making it the most prevalent, followed by breast cancer (11.9%), colorectal cancer (9.7%), and prostate cancer (7.9%). Additionally, 60% of cancer cases and 70% of cancer fatalities occur in Africa, Asia, Central America, and South America, which are the less developed parts of the world [5].

Natural products play a significant role in the development of chemotherapeutic medicines, and they are also thought to be a main source of novel therapies that feed the existing clinical pipeline for treating cancer. Taxanes (e.g. Taxol), vinblastine, vincristine, and the podophyllotoxin are examples of clinically important antitumor drugs originating from higher plants and have largely contributed to the management of human diseases since their discovery early in the 20th century [6–9].

Terrestrial fungi have long been known as a rich source of biologically active secondary metabolites and for treatment of many disorders since the discovery of penicillin by Sir Alexander Fleming 1928, which has led to a breakthrough in the treatment of bacterial infections [10–12]. Endophytic fungi are the hidden members of the microbial community and have received less research interest than their more pathogenic counterparts. Thus, they represent an under-utilized resource in the search for new compounds from unexplored microbes. Despite the large number of anticancer hits identified from fungi, fungal biodiversity has been only partially exploited. It is estimated that only 5% of fungi have been cultured in laboratories [13], this is eventually because of numerous technological, biotechnological, and physiological factors such as the difficulty of the fermentation and cultivation process which is more complicated than plants or bacterial growth. In fact, fungi frequently create promising metabolites in laboratories in milligram quantities, which are typically only sufficient for the preliminary anticancer bioassays, or they are readily available commercially as pricy biochemical reagents [12, 14]. Fungi would thus offer an enormous source of novelty if the limitations of their isolation and culturing could be overcome [15, 16].

From this viewpoint, our study aims to describe the isolation and identification of five fungal strains associated with the Red Sea soft coral *Paralemnalia thyrsoides* (Ehrenberg 1834), which is one of the most common marine invertebrates, natively distributed throughout tropical and subtropical regions of the Indo-Pacific Ocean [17]. Octocorals of the genus *Paralemnalia* (family Nephtheidae) represent a rich source of natural metabolites with intriguing and unique chemical features, such as; sesquiterpenoids, norsesquiterpenoids and diterpenoids [17–19]. Moreover, the cytotoxic potential of the fungal ethyl acetate extracts was investigated against four cancer cell lines. In addition, the fungal extracts were also explored by LC–HR-ESI-MS-based metabolomics and the identified compounds were afterwards subjected to *in silico* analysis in order to gain insights into the mechanism of the cytotoxic activity.

## Materials and methods

### Soft coral collection and identification

The soft coral *Paralemnalia thyrsoides* was collected, identified and treated for fungal isolation (see supplementary file).

### Isolation and purification of fungal strains

The soft coral biomass was washed twice with sterile seawater, dried and submerged in 70% ethanol for one to two minutes for surface sterilization and then allow to air dry. Furthermore, the coral interior tissues were divided into tiny pieces measuring 0.5 cm^3^ a piece using sterile scalpel under sterile conditions. On Sabouraud dextrose agar plates (the SDA were dissolved in sea water and supplied with amoxicillin and flucloxacillin 0.05 g/L to inhibit bacterial growth) the tiny segments were surface streaked. The plates were then incubated at 28 °C for up to two weeks and monitored frequently for any growth, and then the hyphal tips of the fungi were removed and transferred to fresh Sabouraud dextrose agar medium. Plates were prepared in duplicates to reduce the possibility of contamination. Repeated subcultures were done until pure isolates were obtained. Morphological identification was done for each isolate [20, 21].

### Molecular identification and phylogenetic analysis

Molecular identification of the isolated fungal strains was achieved by DNA amplification and sequencing of partial 18 S rRNA gene sequences and the fungal internal transcribed spacer (ITS) region [22]. Genomic DNA was extracted from fungal biomass harvested from agar plants MasterPure™ Yeast DNA purification kit (epicentre, Illumina Company) after a mechanical treatment of the bacterial biomass (approx. 500 mg fresh weight) with 0.5 g glass beats in the presence of 1 x PBS buffer pH 7.2, incubation with 1 ml of a 100 mg/ml lysozyme solution (in TE buffer, pH 8.0) at 37 °C for 16 h), and an achromopeptidase treatment (60 U) at 37 °C (30 min). Finally, the DNA was resolved in 40 µL pure water. The DNA quality and quantity were checked using NanoDrop spectrophotometer. Between 10 and 50 ng were used as DNA template per polymerase chain reaction (PCR) which was performed in a volume of 50 µL. The nearly full-length 18 S rRNA gene and the adjacent ITS region including the ITS1, 5.8 S rRNA gene and ITS2 were amplified with the primer system NS1 (5´- GTAGTCATATGCTTGTCTC-3´) and ITS4 (5´-TTCCTCCGCTTATTGATATGC-3´). The front part of the 18 S rRNA genes was sequenced with primer NS1 and the complete ITS region with primer ITS4. A first phylogenetic assignment based on the partial 18 S rRNA gene and ITS region sequence (including ITS1, 5.8 S rRNA gene, and ITS2 sequences) was performed by BLASTn analysis against GenBank nucleotide sequence database and the internal transcribed spacer (ITS) from fungi type and reference strain databases provided in BLASTn tool of NCBI. The partial 18 S rRNA gene sequences of the three strains were added to the SSU database SSURef NR 99 release 138.1 (12.06.2020) created by the SILVA project.

### Cultivation of pure fungal strains and extraction of fungal cultures

The isolated, pure fugal strains have been developed and extracted for cytotoxic activity testing and LC/MS chemical profiling. (Supplementary file)

### Cell culture

MDA-MB-231, A549, CT-26 and U87 cells were purchased from American Type Culture Collection (ATCC) (Manassas, VA, USA). The cells, lung carcinoma (A549) and colorectal carcinoma (CT-26) were cultured in RPMI-1640 supplemented with 1% Pen/Strep (100 U mL^–1^, Gibco, Carlsbad, CA) and 10% FBS (Atlanta Biologicals, Flowery Branch, GA). While breast carcinoma (MDA-MB-231) and glioblastoma cells (U87) were cultured in DMEM medium supplemented with 1% Pen/Strip and 10% FBS. All the cells were incubated at 37 °C and 5% CO_2_ in humidified incubator (Sanyo Scientific Autoflow, Hudson, MA).

### Cell viability assay

A549, CT-26, MDA-MB-231, and U87 cancer cells were tested for their viability and the half maximal inhibitory concentration IC_**50**_ (µg/mL) values were calculated using GraphPad Prism 9 software. (Supplementary file). Cells were grown in 96- well plates at a density of 4 × 10^4^ per well. The control group n = 6 incubated with 100 µL/well of fresh media and solvent control, ethyl acetate while the cells treated with the fungal extracts (RD1, RD2, RD3, RD4 and RD5) incubated with 100 µL/well, n = 6.

After 24 h, the medium was aspirated and replaced with 100 µL of fresh media and 20 µL of MTS reagent in each well (Cell Titer 96 Aqueous One Solution cell proliferation assay, Promega Corporation, Madison, WI, USA). The plates were then incubated at 37 °C with 5% CO_2_ for 2 h. The cells were examined under a cell imaging system (EVOS FL Digital Microscope) using a 20X objective. The absorbance was measured at 490 nm using a Spectramax plus 384 Microplate reader (Molecular Devices, Sunnyvale, CA, USA). Relative cell viability values were expressed as the percentage of absorbance from the treated wells compared to the control wells (untreated), with the control wells’ viability set to 100%. The half maximal inhibitory concentration (IC_50_) values (µg/mL) were obtained using GraphPad Prism 9 software.

### Metabolomics analysis

A Synapt G2 HDMS quadrupole time-of-flight hybrid mass spectrometer and an Acquity Ultra Performance Liquid Chromatography system were used to perform metabolomics profiling on the crude extracts of the fungal cultures. (for details, see Supplementary file)

### In silico molecular docking

Docking was carried out using several enzymes and receptor proteins involved in cell cycle, cell development, and DNA replication in order to preliminary study the probable molecular targets and to confirm the experimental activity testing for these anticancer drugs. (for details, see Supplementary file)

## Results and discussion

### Isolation of associated fungi and phylogenetic analysis

**Fig. 1:**
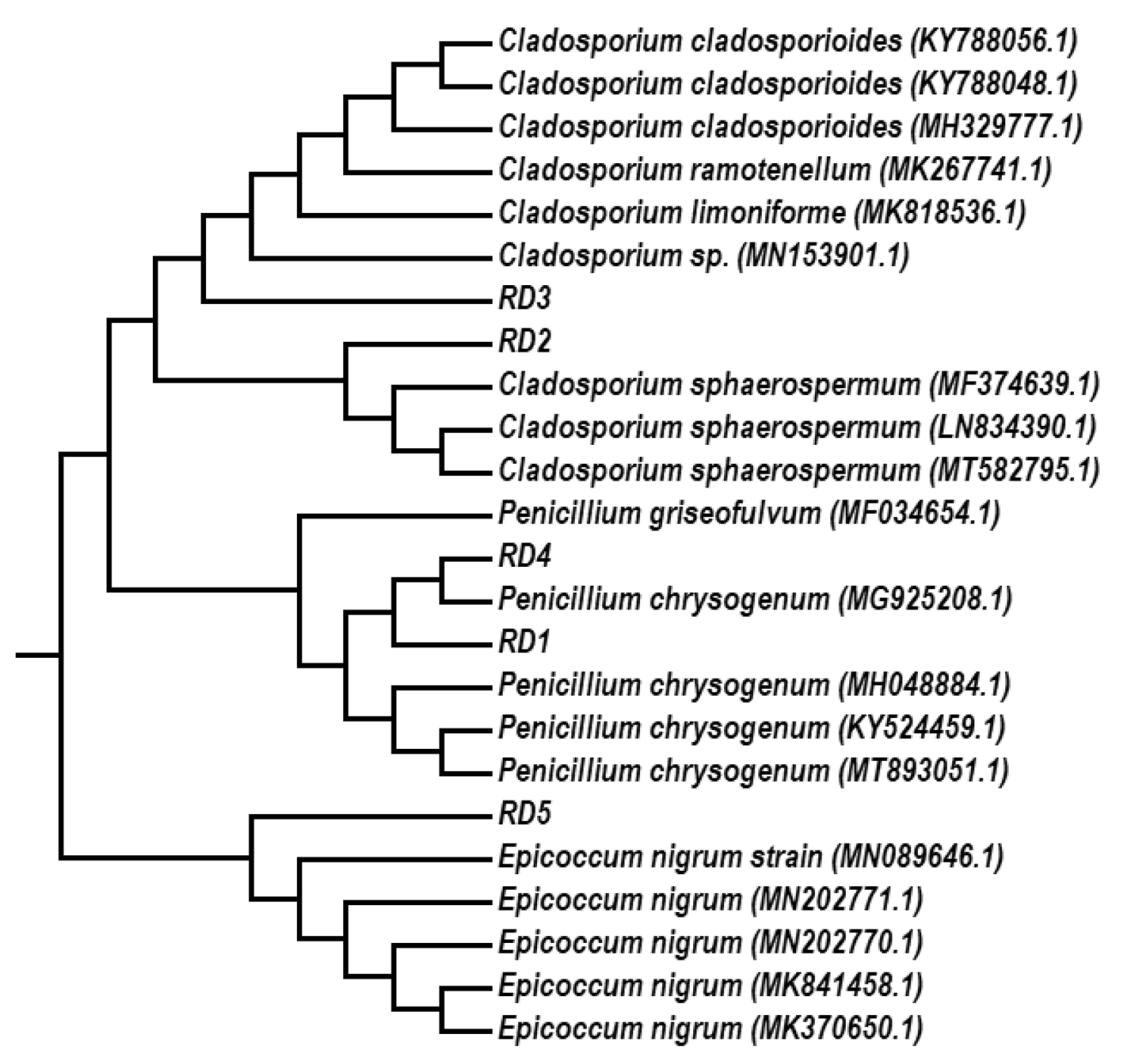
**Evolutionary relationships of taxa.** The evolutionary history was inferred using the UPGMA method [56]. The optimal tree with the sum of branch length = 0.41159388 is shown. The evolutionary distances were computed using the Kimura 2-parameter method [57] and are in the units of the number of base substitutions per site. The analysis involved 24 nucleotide sequences. All positions containing gaps and missing data were eliminated. There was a total of 393 positions in the final dataset. Evolutionary analyses were conducted in MEGA6 [58]

Based on phenotypic characteristics, eight different pure fungal isolates were obtained in this study. Five fungal isolates were selected according to their cultural characteristic for further molecular analysis and to continue the study. From phenotypic characteristics and 18 S rRNA gene and ITS region sequences analysis via the BLASTn tool of the National Center of Biotechnology Information (NCBI); the isolate RD1 was identified to be *Penicillium griseofulvum*, RD2 to be *Cladosporium sphaerospermum*, RD3 to be *Cladosporium liminiforme*, RD4 to be *Penicillium chrysogenum* and finally RD5 to be *Epicoccum nigrum* (Table S1.). The sequences of the genes were deposited to the GenBank database with the accession numbers OQ740602, OQ619183, OQ773545, OQ773633 and OQ780763 for the isolates RD1, RD2, RD3, RD4, and RD5 respectively. The phylogenetic analysis was performed with 15,000 and 2000 bp sequences for *Penicillium griseofulvum*, *Cladosporium sphaerospermum*, *Cladosporium liminiforme*, *Penicillium chrysogenum*, and *Epicoccum nigrum* using MEGA 6 software (Fig. 1).

### Cytotoxic activity

**Fig. 2:**
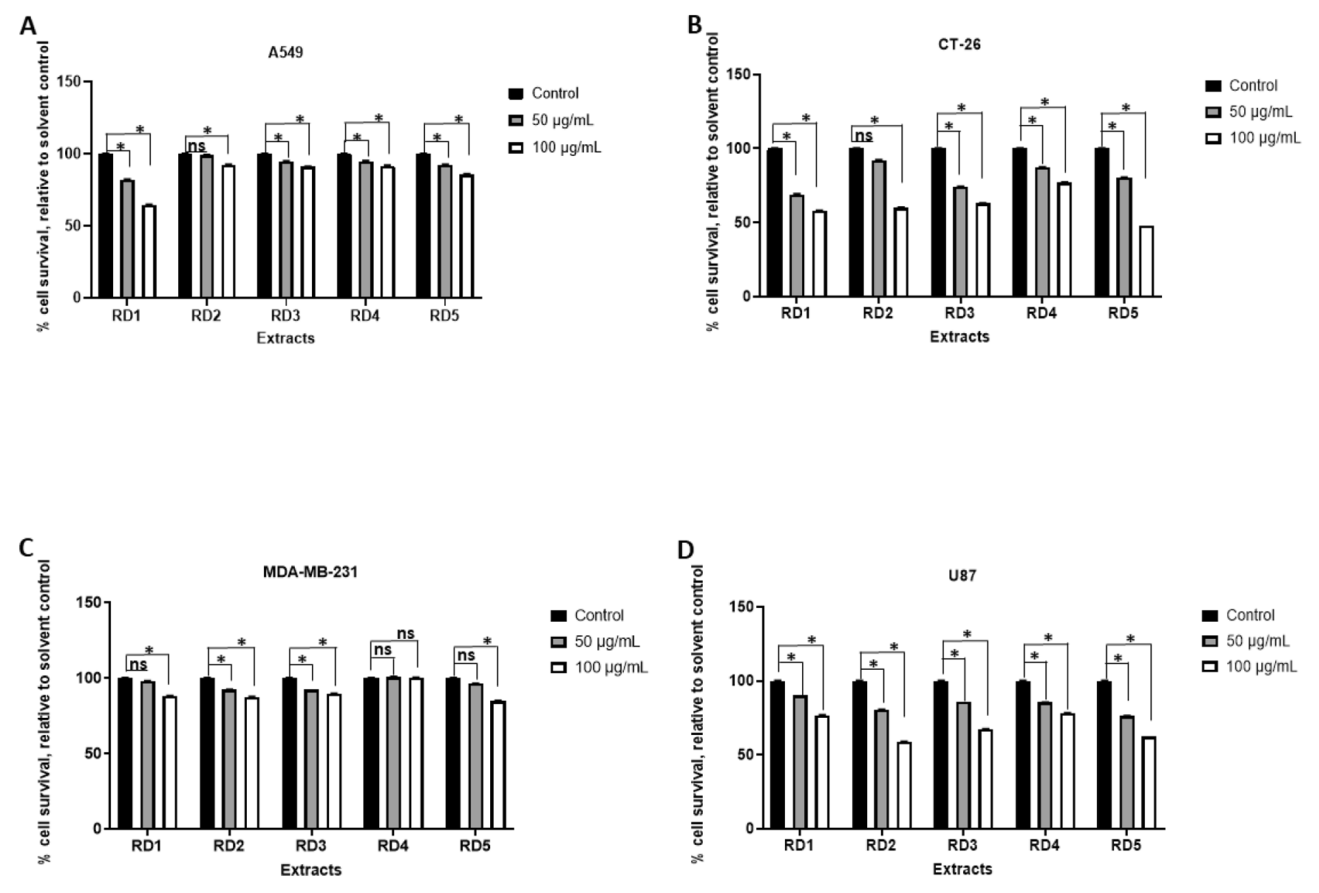
The cytotoxic activities of the five fungal strains RD1, RD2, RD3, RD4 and RD5 were determined using the MTS assay. Cells A549 (**A**), CT-26 (**B**), MDA-MB-231 (**C**) and U87 (**D**) were tested. The graph represents the analysis of six replicates and shows the percentage of survival of A549, CT-26, MDA-MB-231 and U87 - treated cells relative to matching solvent-treated cells (treated with ethyl acetate). All experiments were performed at least two times. *P < 0.05, Student’s *t*-test. n.s., statistically not significant

The in vitro cytotoxic activity of the five fungal isolates RD1, RD2, RD3, RD4, and RD5 were examined on lung, colorectal, breast, and glioblastoma carcinoma cell lines (A549, CT-26, MDA-MB-231, and U87), respectively. These cancer cell lines were incubated with different concentrations of the fungal extracts (50 and 100 µg/mL) for 24 h. Whereas, RD1 fungal extract at 50 µg/mL showed statistically significant effect on A549, CT-26 and U87 cancer cell lines, on the other hand, it didn’t show any significant effect on MDA-MB-231 cell line. Likewise, RD1 extract showed significant effect against the four cancer cell lines at 100 µg/mL. RD2 extract didn’t show statistically significant effect at 50 µg/mL against A549 and CT-26 cell lines, while at 100 µg/mL, it exhibited a significant effect on all tested cancer cell lines. On the other hand, RD3 extract with the two concentrations revealed statistically significant effect against the four cancer cell lines. Likewise, RD4 extract exhibited statistically significant effect with both concentrations against A549, CT-26 and MDA-MB-231, while RD4 didn’t reveal any significant effects against U87. RD5 extract with both concentrations (50 and 100 µg/mL) showed statistically significant effect against A549, CT-26 and U87, while MDA-MB-231 only significantly affected with RD5 at 100 µg/mL **(**Fig. 2). These results suggested that these extracts could be very promising agents for cancer treatment. The 50% cytotoxic effect is considered strongly active at a concentration that is below 10 µg/mL and moderate active compounds are between 11 and 100 µg/ml. As shown in table S2 fungal extract RD3 and RD4 showed strong activity against A549 since the IC_50_ was 6.99 ± 3.47 and 1.45 ± 8.54, respectively (lower than 10 µg/mL). While RD1, RD2 and RD5 extracts showed moderate effect since IC_50_ was 46.73 ± 3.24, 79.20 ± 1.16 and 91.17 ± 4.3, respectively (between 11 and 100 µg/mL). RD4 fungal extract showed strong activity with CT-26 and MDA-MB-231 cancer cell lines compared to RD1, RD2, RD3 and RD5 that showed moderate effect.

According to IC_50_ values all the ethyl acetate extract of the different isolates revealed various and selective cytotoxic activities against the four tested cell lines. In which, RD4 ethyl acetate extract revealed the strongest cytotoxic potency against three cancer cell lines A549, CT-26 and MDA-MB-231 with IC_50_ values 1.45 ± 8.54, 1.58 ± 6.55 and 1.39 ± 2.0 µg/mL, respectively, whereas it showed moderate activity against U87 cell line (IC_50_ 36.28 ± 1.49 µg/mL). Likewise, RD3 revealed selective cytotoxic potency against A549 with IC_50_ 6.99 ± 3.47 µg/mL and moderate activity against MDA-MB-231 cell line (IC_50_ 37.50 ± 7.73 µg/mL). In addition, RD1 extract revealed selected potency against U87 with IC_50_ 5.507 ± 6.79 µg/mL, as indicated in Table S2.

### Metabolomics profiling of the culture extracts

In the field of metabolomics, each organism chemical profile or chemical fingerprint is provided under specific environmental conditions. It is essential for discovering new bioactive metabolites and natural drugs, as well as for enhancing fungal fermentation techniques and controlling the isolation of specific bioactive molecules [23]. The ethyl acetate extracts obtained from the fermentation of the five fungal strains were analyzed in positive and negative ion mode by LC-HR-ESI-MS for dereplication purposes (Figure S1).

**Fig. 3:**
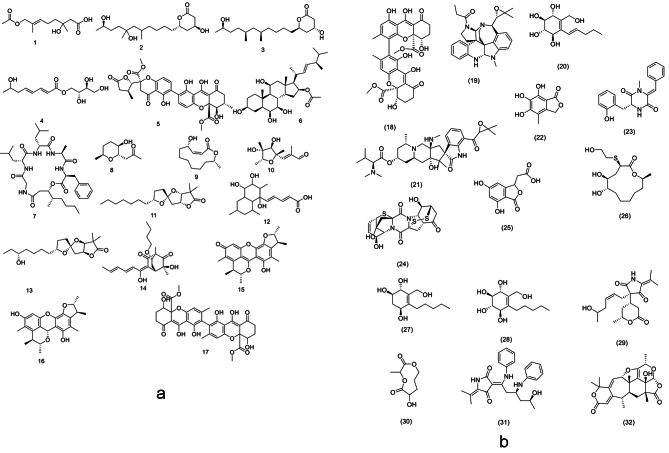
**a.** Chemical structures of the dereplicated metabolites from the ethyl acetate extracts of the five fungal strains (**1–17**). **b.** Chemical structures of the dereplicated metabolites from the ethyl acetate extracts of the five fungal strains (**18–32**)

The annotated compounds (Table S3, Fig. 3a &b) belonged to multivariate classes of secondary metabolites such as alkaloids, terpenoids, phenolic derivatives, and benzenoids. In this context, the mass ion peaks at *m/z* [M-H]^−^ 243.123 (RT, 3.891 min), 329.232 (RT, 5.769 min) and 313.237 (RT, 6.609 min), were identified as a monoterpene Penicimonoterpene **(1)**, polyketide derivatives penicitide B **(2)** and penicitide A **(3)**, corresponding to the molecular formulas C_12_H_20_O_5_, C_18_H_34_O_5_ and C_18_H_34_O_4_. In addition, the mass ion peak at *m/z* 259.118 [M-H]^−^ (RT, 3.853 min), in conformity with the predicted molecular formula C_12_H_20_O_6_, was characterized as penicierythritols B **(4)**. The mass ion peak at *m/z* 637.155 [M-H]^−^ (RT, 6.305 min) for the suggested molecular formula C_32_H_30_O_14_ was recognized as chrysoxanthone A **(5)**. Moreover, the mass ion peak *m/z* 505.351 [M-H]^−^ (RT, 9.627 min), in consonance with the predicted molecular formula C_30_H_50_O_6_ was identified as penicisteroid A **(6)**. Besides, the metabolite, namely chrysogeamide E **(7)** with molecular formula C_35_H_55_N_5_O_7_ was dereplicated from the mass ion peak at *m/z* 656.401 [M-H]^−^ (RT, 8.431 min). The metabolites **1**–**7** were found to be previously isolated from *Penicillium chrysogenum* [24–28]. Additionally, another metabolite with molecular formula C_9_H_16_O_3_ was dereplicated from the mass ion peak at *m/z* 171.102 [M-H]^−^ (RT, 5.672 min) as decarestrictine L **(8)**, it was formerly obtained from *Penicillium simplicissimum* [29]. Furthermore, the mass ion peak at *m/z* [M + H] ^+^ 213.148 (RT, 5.308 min), corresponding to the molecular formula C_12_H_20_O_3_ was identified as patulolide C **(9)** which was earlier isolated from *Penicillium urticae* [30]. Another metabolite dereplicated from the mass ion peak at *m/z* 213.112 [M-H]^−^ (RT, 4.418 min) in accordance with the molecular formula C_11_H_18_O_4_, was identified as citreoviral **(10)** and was previously reported from *Penicillium citreoviride* [31]. Likewise, the mass ion peaks at *m/z* 309.206 and 323.186 [M-H]^−^ (RT, 5.364 and 7.807 min) corresponding to the molecular formulas C_18_H_30_O_4_ and C_18_H_28_O_5_ were characterized as cephalosporolide H **(11)** and hynapene A **(12)**, which were found to be also isolated from *Penicillium* sp. [32, 33]. The molecular formulas C_18_H_30_O_5_ and C_20_H_28_O_5_ were dereplicated from the mass ion peaks at *m/z* 327.216 and 349.200 [M + H] ^+^ (RT, 5.325 and 5.292 min) and were identified as penisporolide A **(13)** and rezishanone B **(14)**, they were reported to be isolated from *Penicillium* sp. [34] and *Penicillium notatum* [35], respectively. Similarly, penicitrinone A **(15)** and penicitrinol A **(16)**, which have been formerly isolated from *Penicillium citrinum* [36], were dereplicated from the mass ion peaks at *m/z* 379.158 and 381.161 [M-H]^−^ (RT, 7.725 and 7.731 min) in compliance with the molecular formulas C_23_H_24_O_5_ and C_23_H_26_O_5_. Another mass ion peak at *m/z* 637.155 [M-H]^−^ (RT, 6.305 min), in conformity with the molecular formula C_32_H_30_O_14_, was identified as rugulotrosin A **(17)** and rugulotrosin B **(18)**, also, previously isolated from *Penicillium* sp. [37]. Furthermore, the mass ion peak at *m/z* 469.261 [M-H]^−^ (RT, 7.014 min) for the predicted molecular formula C_29_H_34_N_4_O_2_ was distinguished as communesin G **(19)** that was formerly obtained from *Penicillium rivulum* [38]. In addition, the mass ion peak at *m/z* 427.175 [M-H]^−^ (RT, 8.107 min) for the molecular formula C_24_H_28_O_7_ was dereplicated as paraherquonin **(20)**, which has been reported from *Penicillium paraherquei* [39]. Whereas, the metabolite citrinadin A **(21)** with the molecular formula C_35_H_52_N_4_O_6_, was previously purified from *Penicillium citrinum* [40], was characterized from the mass ion peak at *m/z* 625.396[M + H]^+^ (RT, 7.758 min).

In addition to the above mentioned metabolites, the metabolomics analysis revealed the presence of three metabolites, were reported in *Epicoccum nigrum* fungus and dereplicated from the mass ion peaks at *m/z* 239.058, 321.124, and 453.025 [M-H]^−^ (RT, 8.864, 3.906, and 3.465 min). These metabolites were identified as (+)- epicoccone C **(22)** [41], (±)-5 hydroxydiphenylalazine A **(23)** [42], and amphiepicoccin I **(24)** [43], matched with the molecular formulas C_11_H_12_O_6_, C_19_H_18_N_2_O_3,_ and C_18_H_18_N_2_O_6_S_3_, respectively. On the other hand, metabolites dereplicated from *Cladosporium sp.* included one phthalide compound that was identified from the mass ion peak at *m/z* 223.026 [M-H]^−^ (RT, 7.768 min) in agreement with the molecular formula C_10_H_8_O_6_, it was reported to be isolated from the fungus *Cladosporium herbarum* as herbaric acid **(25)** [44]. One macrolide, characterized as thiocladospolide E **(26)**, earlier purified from *Cladosporium* sp. *SCNU-F0001* [45], was dereplicated from the mass ion peak at *m/z* 305.150 [M-H]^−^ (RT, 4.251 min) in consonance with the molecular formula C_14_H_26_O_5_S. Three cyclohexene derivatives, cladoscyclitol A **(32)**, cladoscyclitol C **(27)**, and cladoscyclitol D **(28)** were reported to be isolated from *Cladosporium* sp. *JJM22* [46] and dereplicated from the molecular ion peaks at *m/z* 243.123, 229.144, and 245.138 [M-H]^−^ (RT, 3.891, 4.004, and 4.8498 min) in compliance with the molecular formulas with molecular formula C_12_H_20_O_5_, C_12_H_22_O_4_, and C_12_H_22_O_5_, respectively. Furthermore, two tetramic acids, cladosporiumin B **(29)** and (*Z*)-cladosin K **(31)** were dereplicated from the mass ion peaks at *m/z* 350.202 [M + H] ^+^ (RT, 5.228 min) and at *m/z* 418.208 [M-H]^−^ (RT, 6.084 min) corresponding to the molecular formulas C_19_H_27_NO_5_ and C_25_H_29_N_3_O_3_. They were isolated from *Cladosporium* sp. *SCSIO z0025* [47] and *Cladosporium sphaerospermum* [48], respectively. The last metabolite was identified from the mass ion peak *m/z* 227.128 [M-H]^−^ (RT, 6.250 min) in match with the molecular formula C_12_H_20_O_4_, it was previously isolated from *Cladosporium* sp. *TZP29* [49] and was identified as cladospolide E **(30)**.

### In silico molecular docking

**Fig. 4:**
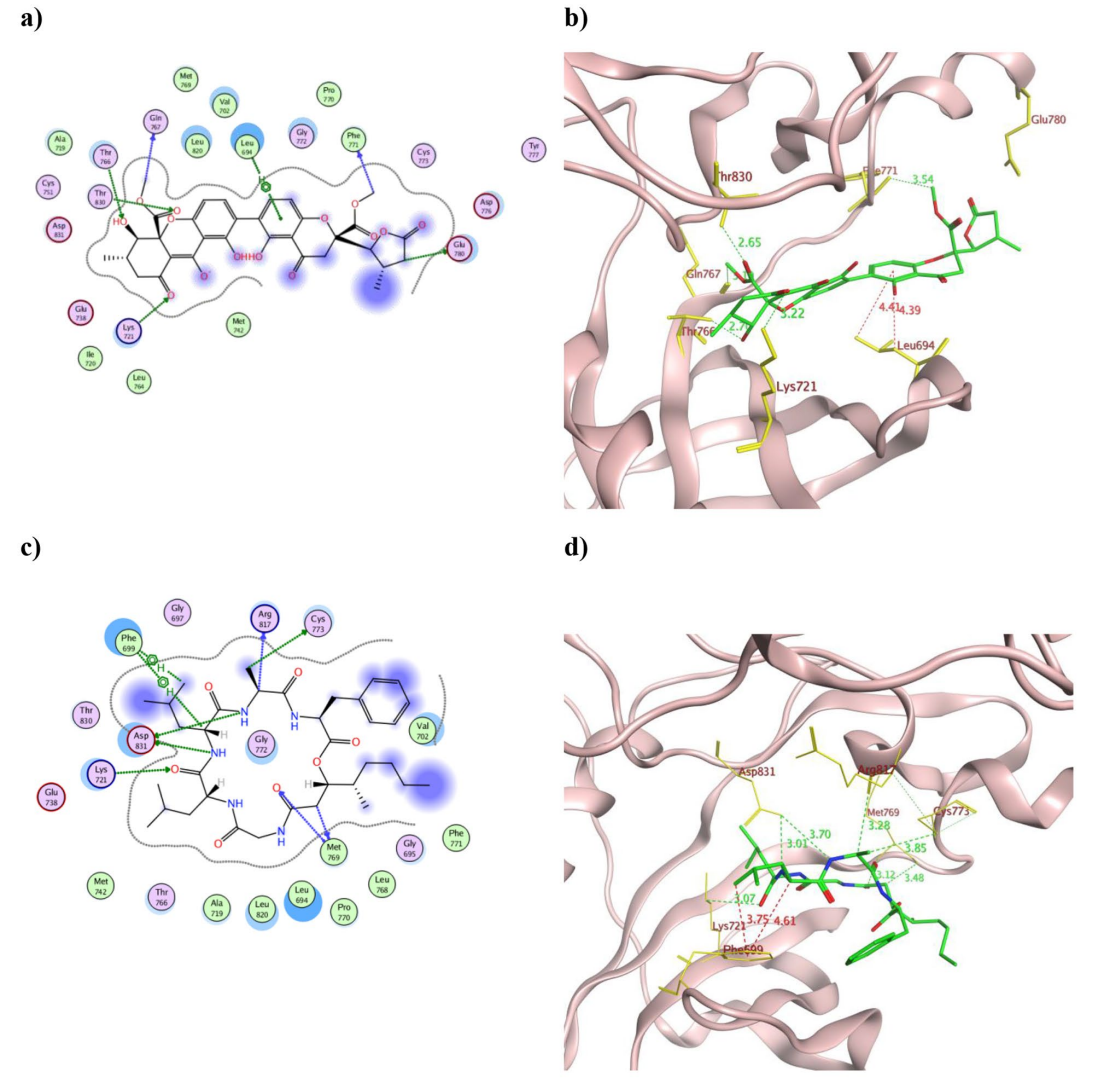
The 2D and 3D presentation of the binding conformations of the isolated compounds chrysoxanthone A **5** (a and b) and chrysogeamide E **7** (c and d) using EGFR (PDB: 1M17) where the tested compound appeared as green stick model connected to the pocket through H- and hydrophobic bonds displayed as green and red dotted lines, respectively with their corresponding distance in Å

**Fig. 5:**
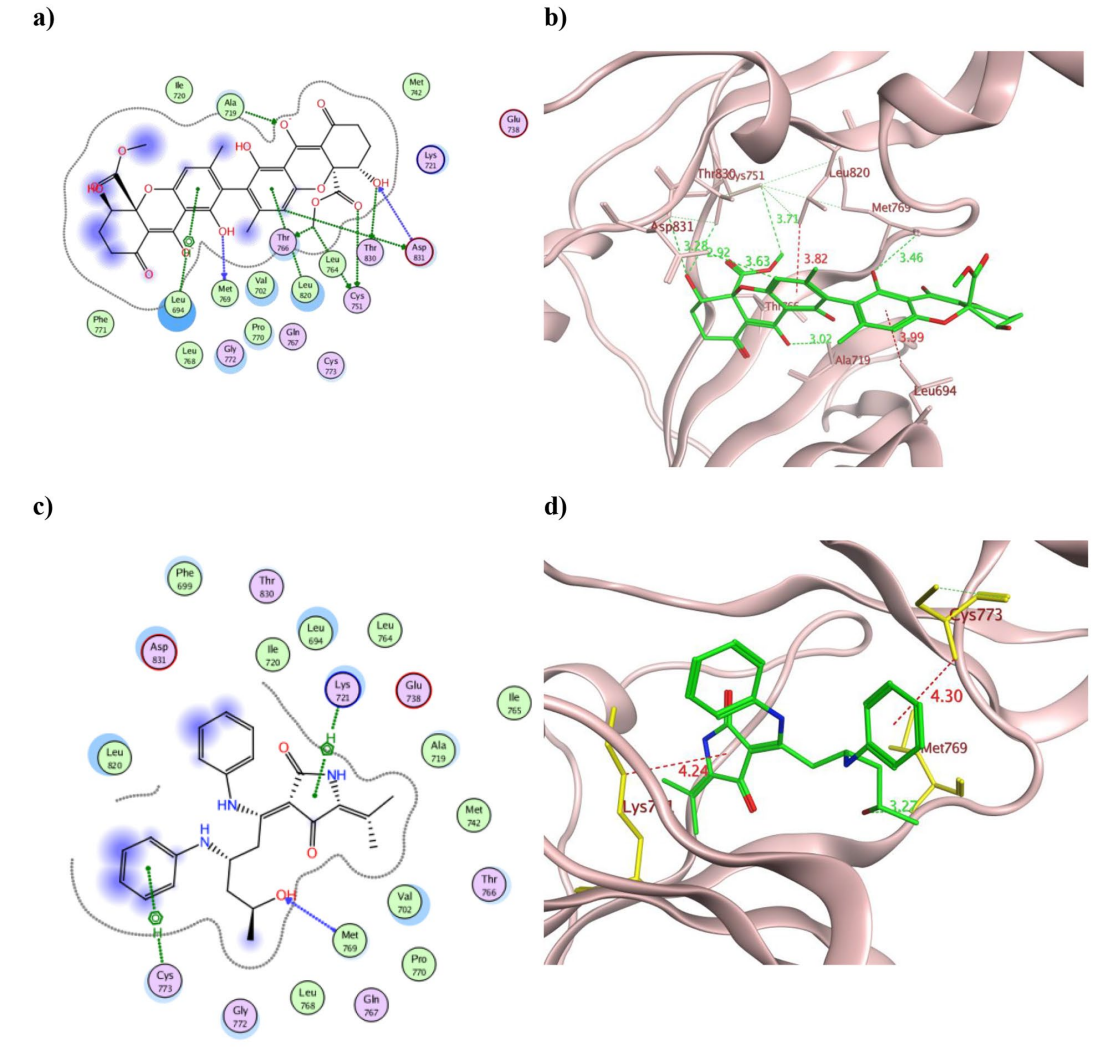
The 2D and 3D presentation of the binding conformations of the isolated compounds rugulotrosin A **17** (a and b) and (*Z*)-cladosin K **31** (c and d) using EGFR (PDB: 1M17) where the tested compounds appeared as green stick model connected to the pocket through H- and hydrophobic bonds displayed as green and red dotted lines, respectively with their corresponding distance in Å

**Fig. 6:**
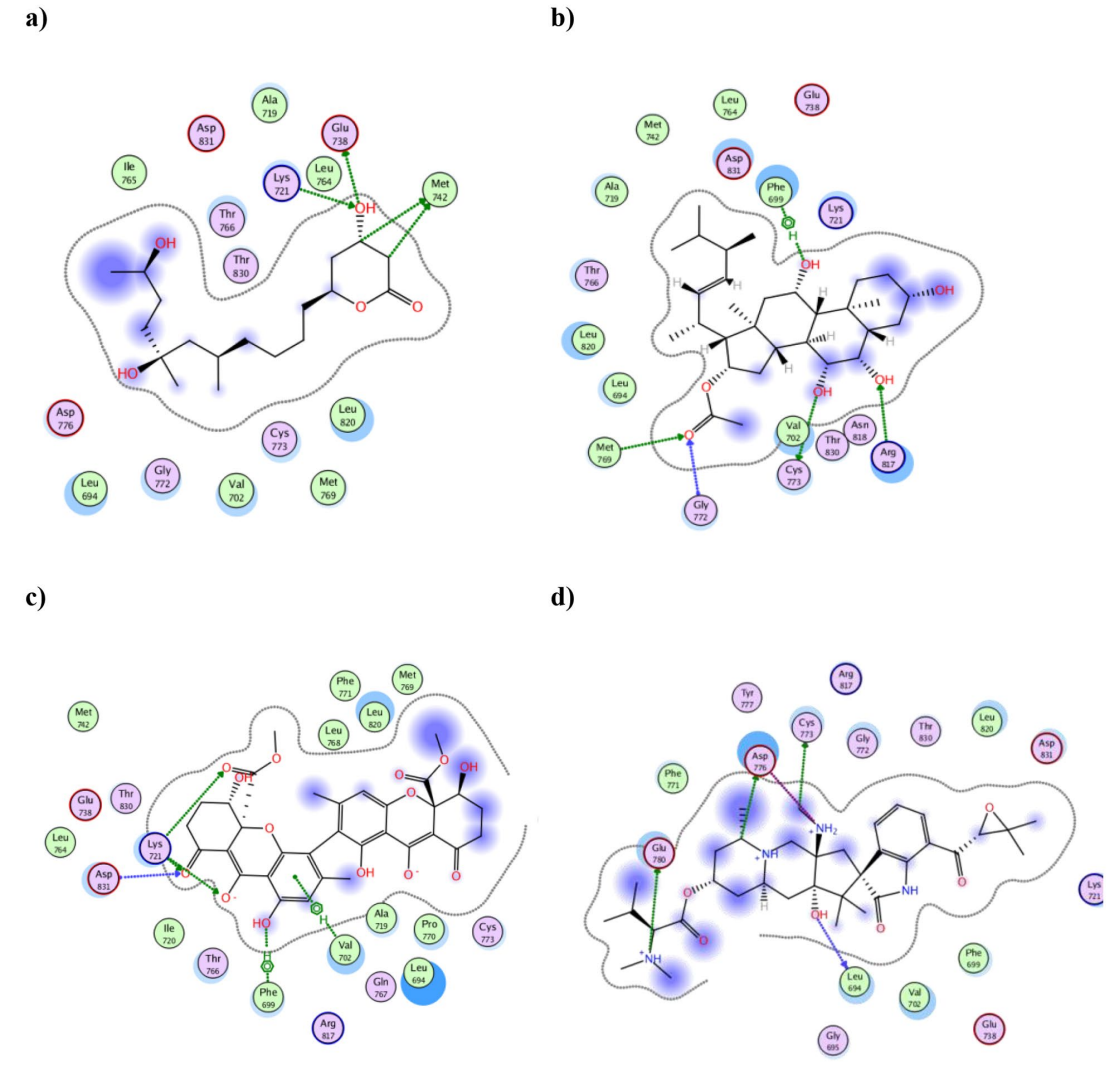
The 2D presentation of the binding interactions of penicitide B **(2)**, penicisteroid A **(6)**, rugulotrosin B **(18)** and citrinadin A **(21)** with EGFR (PDB:1M17)

To better understand the pharmacological mechanism of the fungal extracts in inhibiting the tested cancerous cell lines, their dereplicated compounds were *in silico* evaluated against the Epidermal Growth Factor Receptor tyrosine kinase (EGFR). The molecular docking results for the 32 dereplicated compounds were presented in (Table S4) and the 2D as well as the 3D conformations of the best fitted derivatives were illustrated in Figs. 4, 5 and 6.

EGFR is widely distributed in the cell membrane and normally modulates the cell proliferation and differentiation. Nonetheless, its overexpression was detected in many types of solid tumors [50, 51]. EGFR has dual properties as a receptor with its extra-cellular domain and a kinase enzyme intra-cellular domain where the ATP-binding site locates. Both domains are connected through the trans-membranal domain and usually presents in a monomeric form during its resting state [52, 53]. Structurally, its ATP-binding domain has a conserved amino acid sequence with 39 residues locate near its binding site among which Leu718, Val726, Ala743, Met793, and Leu844 showed abundant ligand interaction [54]. During the activation phase, Lys721 forms ion-pair with the conserved Glu738 to interact with the ATP phosphate groups [55]. As depicted from Table S4, most of the isolated compounds managed to bind to one or more of the crucial amino acid residues at EGFR substrate binding site and/or its ATP-binding site such as Lys721, Glu738 and Met769. The best binding energy scores were demonstrated by chrysoxanthone A **(5)**, chrysogeamide E **(7)**, rugulotrosin A **(17)** and (*Z*)-cladosin K **(31)** revealing − 10.33, -11.66, -10.51 and − 9.79 Kcal/mol, respectively that surpassed the co-crystallized ligand AQ4 (-9.52 (Kcal/mol). Moreover, the carbonyl of chrysoxanthone A **(5**) managed to form an ionic bond with Lys721 with a distance of 3.22 Å and additional H-bond with Gln767 that resembled AQ4 (Fig. 4a and b). Furthermore, the phenyl ring of compound **5** formed two pi -H interactions with Leu694 to maintain its binding conformation. Other H-bonds were observed between **5** and Thr766, Phe771, Glu780, and Thr830 at EGFR binding site (Fig. 4a). On the other hand, chrysogeamide E **(7)** exhibited the best binding energy to EGFR and was capable to interact with several amino acid residues through hydrogen bonds such as Lys721, Met769, Cys773, Arg817 and Asp831 in addition to two H-pi interaction with Phe699 (Fig. 4c). The formed H-bonds with the crucial Met769 were of distance 3.12 Å and 3.48 Å while in case of Lys721 was 3.07 Å (Fig. 4d). Similarly, the phenolic hydroxyl group in compound **(17)** bound to the crucial Met769 as well as extensive H-bonds formation with Ala719, Cys751, Thr766, Leu821, Asp831 and Thr830 (Fig. 5a and b). Besides, the two phenyl moieties of rugulotrosin A **(17)** formed hydrophobic interactions with both Leu694 and Leu820 that supported its tight binding with EGFR. In a similar way, the hydroxyl group in compound (**31)** formed H-bond with Met769 of 3.27 Å distance and hydrophobic interactions with the crucial Lys721 and Cys773 by its 6-membered and 5-membered rings, respectively (Fig. 5c and d).

Among the compounds with comparable binding energy scores to the co-crystallized ligand AQ4 were penicitide B **(2)**, penicisteroid A **(6)**, rugulotrosin B **(18)** and citrinadin A **(21)** that showed − 9.16, -9.11, -9.66 and − 9.53 Kcal/mol, respectively. Their 2D interactions with EGFR binding site revealed multiple interactions through hydrogen and ionic bonding with the bind site residues (Fig. 6). The hydroxyl group of **2** fashioned two strong H- bonds with Lys721 and Glu738 of distances 3.02 Å and 2.75 Å, respectively (Fig. 6a). The polar groups of **6** produced four H-bonds with Gly772, Met769, Cys773 and Arg817 beside one of its hydroxyl functionality formed pi-H with Phe699 (Fig. 6b). Likewise, strong ionic interaction was observed between the ionized hydroxyl group of **18** and Lys721 in addition to a second H-bond with the side ester (Fig. 6c). Furthermore, two additional hydrophobic interactions were spotted with Phe699 and Val702. Two of the protonated amine moieties of citrinadin A **(21)** bound to Asp776 and Glu780 with an average distance of 3.05 Å as well as other H- bond formation with Leu694 and Cys773 (Fig. 6d).

## Conclusion

The current work revealed the identification and purification of five fungal isolates from Red Sea soft coral, *Paralemnalia thyrsoides*, which were molecularly identified as *Penicillium griseofulvum* (RD1), *Cladosporium sphaerospermum* (RD2), *Cladosporium liminiforme* (RD3), *Penicillium chrysogenum* (RD4), *and Epicoccum nigrum* (RD5). The in vitro cytotoxic assay of the ethyl acetate extracts of the five different fungal strains revealed a significant effect in killing cancer cells, as the RD4 fungal extract showed the strongest potency with IC_50_ values of 1.45 ± 8.54, 1.58 ± 6.55 and 1.39 ± 2.0 µg/mL against the three cancer cell lines A549, CT-26 and MDA-MB-231. Likewise, RD3 revealed selective cytotoxic potency against A549 with IC_50_ value of 6.99 ± 3.47 µg/mL and moderate activity against MDA-MB-231 cell line (IC_50_ 37.50 ± 7.73 µg/mL). Interestingly, in an attempt to unveil the chemical profile of the soft coral associated fungi, the extracts were subjected to LC-HR-ESI-MS based metabolomics analysis, leading to the characterization of 32 metabolites predominantly alkaloids, terpenoids, phenolic derivatives and benzenoids. Additionally, Molecular docking analysis of the dereplicated secondary metabolites was accomplished to explain the mechanism of the fungal extracts in inhibiting the tested cancerous cell lines. The study suggested that EGFR tyrosine kinase inhibitory potential is a plausible mechanism for their cytotoxic activities, in particular chrysoxanthone A (**5**), chrysogeamide E (**7**), rugulotrosin A (**17**) and (Z)-cladosin K (**31**), which displayed exceptional binding abilities with EGFR tyrosine kinase. Our study highlighted the importance of *Paralemnalia thyrsoides*-associated fungi as a promising source of antitumor compounds. Marine fungal endophytes have drawn numerous researchers’ attention in basic and applied research as a distinguished reservoir of bioactive metabolites for the development of diverse natural cytotoxic medications. Nevertheless, further investigations could be dedicated for in-depth understanding of the cytotoxic activity of the extracts and metabolites isolated from these microorganisms.

### Electronic supplementary material

Below is the link to the electronic supplementary material.

Supplementary Material 1

## Data Availability

All data generated or analyzed during this study are included in this published article (and its supplementary information files).

## References

[1] Kharwar RN, Mishra A, Gond SK, Stierle A, Stierle D (2011). Anticancer compounds derived from fungal endophytes: their importance and future challenges. Nat Prod Rep.

[2] Sung H, Ferlay J, Siegel RL, Laversanne M, Soerjomataram I, Jemal A (2021). Global cancer statistics 2020: GLOBOCAN estimates of incidence and mortality worldwide for 36 cancers in 185 countries. Cancer J Clin.

[3] Khazaal HT, Khazaal MT, Abdel-Razek AS, Hamed AA, Ebrahim HY, Ibrahim RR (2023). Antimicrobial, antiproliferative activities and molecular docking of metabolites from Alternaria alternata. AMB Express.

[4] Gulland A. Global cancer prevalence is growing at “alarming pace,” says WHO. In.: British Medical Journal Publishing Group; 2014. 10.1136/bmj.g1338 24496041

[5] Gomes NG, Lefranc F, Kijjoa A, Kiss R (2015). Can some marine-derived fungal metabolites become actual anticancer agents?. Mar Drugs.

[6] Habtemariam S, Lentini G (2018). Plant-derived anticancer agents: lessons from the pharmacology of geniposide and its aglycone, genipin. Biomedicines.

[7] Hussain A, Bourguet-Kondracki M-L, Majeed M, Ibrahim M, Imran M, Yang X-W (2023). Marine life as a source for breast cancer treatment: a comprehensive review. Biomed Pharmacother.

[8] Moraes DFC, Mesquita LSSd A, FMMd SR, MNd, Malik S. Anticancer drugs from plants. Biotechnology and production of anti-cancer compounds. Springer; 2017. 121–42.

[9] Carroll AR, Copp BR, Davis RA, Keyzers RA, Prinsep MR. Marine natural products. Nat Prod Rep. 2022. 10.1039/d1np00076d 35201245

[10] Kjer J, Debbab A, Aly AH, Proksch P (2010). Methods for isolation of marine-derived endophytic fungi and their bioactive secondary products. Nat Protoc.

[11] Bakhtra D, Yanwirasti Y, Wahyuni FS, Aminah I, Handayani D (2022). Antimicrobial and cytotoxic activities screening of Marine Invertebrate-Derived Fungi Extract from West Sumatera, Indonesia. Open Access Macedonian Journal of Medical Sciences.

[12] Gupta A, Meshram V, Gupta M, Goyal S, Qureshi KA, Jaremko M (2023). Fungal endophytes: microfactories of Novel Bioactive Compounds with therapeutic interventions; a Comprehensive Review on the Biotechnological Developments in the field of Fungal Endophytic Biology over the last decade. Biomolecules.

[13] Demain AL (2006). From natural products discovery to commercialization: a success story. J Ind Microbiol Biotechnol.

[14] Kornienko A, Evidente A, Vurro M, Mathieu V, Cimmino A, Evidente M (2015). Toward a cancer drug of fungal origin. Med Res Rev.

[15] Rappé MS, Giovannoni SJ (2003). The uncultured microbial majority. Annu Rev Microbiol.

[16] Stevenson BS, Eichorst SA, Wertz JT, Schmidt TM, Breznak JA (2004). New strategies for cultivation and detection of previously uncultured microbes. Appl Environ Microbiol.

[17] Phan GH, Hu H-C, Chang F-R, Wen Z-H, Chen J-J, Chung H-M (2022). Norsesquiterpenoids from the octocoral paralemnalia thyrsoides (Ehrenberg 1834). RSC Adv.

[18] Huang H-C, Ahmed AF, Dai C-F, Sheu J-H. Paraflexinols A – G, rare flexibilane-based diterpenoids from the soft coral Paralemnalia thyrsoides. Tetrahedron. 2023:133561.

[19] Han X, Wang Q, Luo X, Tang X, Wang Z, Zhang D (2021). Lemnalemnanes A–C, three Rare rearranged sesquiterpenoids from the Soft Corals Paralemnalia thyrsoides and Lemnalia sp. Org Lett.

[20] Abdelwahab MF, Fouad MA, Kamel MS, Özkaya FC, Kalscheuer R, Müller WE (2018). Tanzawaic acid derivatives from freshwater sediment-derived fungus Penicillium sp. Fitoterapia.

[21] Manilal A, Sabarathnam B, Kiran G, Sujith S, Shakir C, Selvin J (2010). Antagonistic potentials of marine sponge associated fungi Aspergillus clavatus MFD15. Asian J Med Sci.

[22] Sayed AM, Sherif NH, El-Gendy AO, Shamikh YI, Ali AT, Attia EZ et al. Metabolomic profiling and antioxidant potential of three fungal endophytes derived from Artemisia annua and Medicago sativa. Nat Prod Res. 2020:1–5. 10.1080/14786419.2020.1831495 33043694

[23] Tawfike AF, Tate R, Abbott G, Young L, Viegelmann C, Schumacher M (2017). Metabolomic tools to assess the chemistry and bioactivity of endophytic aspergillus strain. Chem Biodivers.

[24] Gao S-S, Li X-M, Du F-Y, Li C-S, Proksch P, Wang B-G (2010). Secondary metabolites from a marine-derived endophytic fungus Penicillium chrysogenum QEN-24S. Mar Drugs.

[25] Xu K, Wei X-L, Xue L, Zhang Z-F, Zhang P (2020). Antimicrobial meroterpenoids and erythritol derivatives isolated from the marine-algal-derived endophytic fungus Penicillium chrysogenum XNM-12. Mar Drugs.

[26] Gao S-S, Li X-M, Li C-S, Proksch P, Wang B-G (2011). Penicisteroids A and B, antifungal and cytotoxic polyoxygenated steroids from the marine alga-derived endophytic fungus Penicillium chrysogenum QEN-24S. Bioorg Med Chem Lett.

[27] Hou X-M, Li Y-Y, Shi Y-W, Fang Y-W, Chao R, Gu Y-C (2019). Integrating molecular networking and 1H NMR to target the isolation of chrysogeamides from a library of marine-derived Penicillium fungi. J Org Chem.

[28] Zhen X, Gong T, Wen Y-H, Yan D-J, Chen J-J, Zhu P (2018). Chrysoxanthones A–C, three new xanthone–chromanone heterdimers from sponge-associated Penicillium chrysogenum HLS111 treated with histone deacetylase inhibitor. Mar Drugs.

[29] GRABLEY S, HAMMANN P, HÜTTER K, KIRSCH R, KLUGE H, THIERICKE R (1992). SECONDARY METABOLITES BY CHEMICAL SCREENING. 20 DECARESTRICTINES, A NEW FAMILY OF INHIBITORS OF CHOLESTEROL BIOSYNTHESIS FROM PENICILLIUM: III. DECARESTRICTINES E TO M. J Antibiot.

[30] RODPHAYA D, SEKIGUCHI J, YAMADA Y (1986). New macrolides from Penicillium urticae mutant S11R59. J Antibiot.

[31] Shizuri Y, Nishiyama S, Imai D, Yamamura S, Furukawa H, Kawai K (1984). Isolation and stereostructures of citreoviral, citreodiol, and epicitreodiol. Tetrahedron Lett.

[32] Li X, Yao Y, Zheng Y, Sattler I, Lin W, Cephalosporolides H (2007). I, two novel lactones from a marine-derived fungus, penicillium sp. Arch Pharm Res.

[33] Tabata N, Tomoda H, Masuma R, Haneda K, Iwai Y, Omura S, Hynapenes A (1993). B and C, new anticoccidial agents produced by Penicillium sp. I. Production, isolation and physico-chemical and biological properties. J Antibiot.

[34] Li X, Sattler I, Lin W (2007). Penisporolides A and B, two new spiral lactones from the marine-derived fungus Penicillium sp. J Antibiot.

[35] Maskey RP, Grün-Wollny I, Laatsch H (2005). Sorbicillin analogues and related dimeric compounds from Penicillium n otatum. J Nat Prod.

[36] Wakana D, Hosoe T, Itabashi T, Okada K, de Campos Takaki GM, Yaguchi T (2006). New citrinin derivatives isolated from Penicillium citrinum. J Nat Med.

[37] Stewart M, Capon RJ, White JM, Lacey E, Tennant S, Gill JH (2004). Rugulotrosins a and B: two new antibacterial metabolites from an australian isolate of a Penicillium sp. J Nat Prod.

[38] Dalsgaard PW, Blunt JW, Munro MH, Frisvad JC, Christophersen C, Communesins G (2005). New Alkaloids from the psychrotolerant Fungus Penicillium r ivulum. J Nat Prod.

[39] Okuyama E, Yamazaki M, Kobayashi K, Sakurai T (1983). Paraherquonin, a new meroterpenoid from Penicillium paraherquei. Tetrahedron Lett.

[40] Tsuda M, Kasai Y, Komatsu K, Sone T, Tanaka M, Mikami Y (2004). Citrinadin A, a Novel Pentacyclic Alkaloid from Marine-Derived Fungus Penicillium c itrinum. Org Lett.

[41] Yan Z, Huang C, Guo H, Zheng S, He J, Lin J (2020). Isobenzofuranone monomer and dimer derivatives from the mangrove endophytic fungus Epicoccum nigrum SCNU-F0002 possess α-glucosidase inhibitory and antioxidant activity. Bioorg Chem.

[42] Chi L-P, Li X-M, Li L, Li X, Wang B-G (2020). Cytotoxic thiodiketopiperazine derivatives from the deep sea-derived fungus Epicoccum nigrum SD-388. Mar Drugs.

[43] Wang Q, Zhang K, Wang W, Zhang G, Zhu T, Che Q (2020). Amphiepicoccins A–J: epipolythiodioxopiperazines from the fish-gill-derived fungus Epicoccum nigrum HDN17-88. J Nat Prod.

[44] Jadulco R, Brauers G, Edrada RA, Ebel R, Wray V, Sudarsono a (2002). New metabolites from sponge-derived fungi curvularia l unata and cladosporium h erbarum. J Nat Prod.

[45] Huang C, Chen T, Yan Z, Guo H, Hou X, Jiang L (2019). Thiocladospolide E and cladospamide A, novel 12-membered macrolide and macrolide lactam from mangrove endophytic fungus Cladosporium sp. SCNU-F0001. Fitoterapia.

[46] Zhang B, Wu J-T, Zheng C-J, Zhou X-M, Yu Z-X, Li W-S (2021). Bioactive cyclohexene derivatives from a mangrove-derived fungus Cladosporium sp. JJM22. Fitoterapia.

[47] Huang Z-h, Nong X-h, Liang X, Qi S-h (2018). New tetramic acid derivatives from the deep-sea-derived fungus Cladosporium sp. SCSIO z0025. Tetrahedron.

[48] Zhang Z, He X, Wu G, Liu C, Lu C, Gu Q (2018). Aniline-tetramic acids from the deep-sea-derived fungus Cladosporium sphaerospermum L3P3 cultured with the HDAC inhibitor SAHA. J Nat Prod.

[49] Zhu M, Gao H, Wu C, Zhu T, Che Q, Gu Q (2015). Lipid-lowering polyketides from a soft coral-derived fungus Cladosporium sp. TZP29. Bioorg Med Chem Lett.

[50] Guardiola S, Sanchez-Navarro MVM (2019). A third shot at EGFR: New Opportunities in Cancer Therapy. Trends Pharmacol Sci.

[51] Citri A (2006). EGF-ERBB signalling: towards the Systems Level. Nat Rev Mol Cell Biol.

[52] Ogiso H, Nureki RIO (2002). Crystal structure of the complex of human epidermal growth factor and receptor extracellular domains. Cell.

[53] Chlessinger J (2002). Ligand-induced, receptor-mediated dimerization and activation of EGF receptor. Cell.

[54] Zhao Z (2019). Structural insights into characterizing binding Sites in epidermal growth factor receptor kinase. Mutants J Chem Inf Model.

[55] Martin-Fernandez ML, Roberts DTCSK. Structure and dynamics of the EGF receptor as revealed by experiments and simulations and its relevance to non-small cell lung cancer. Cells 2019;8(316). 10.3390/cells8040316 PMC6523254 30959819

[56] Sneath PH, Sokal RR. Numerical taxonomy. The principles and practice of numerical classification. 1973.

[57] Kimura M (1980). A simple method for estimating evolutionary rates of base substitutions through comparative studies of nucleotide sequences. J Mol Evol.

[58] Tamura K, Stecher G, Peterson D, Filipski A, Kumar S (2013). MEGA6: molecular evolutionary genetics analysis version 6.0. Mol Biol Evol.

