# Supplementary material for: Paralemnalia thyrsoides-associated fungi: phylogenetic diversity, cytotoxic potential, metabolomic profiling and docking analysis
Source: BMC Microbiol. 2023 Oct 26;23:308. doi: 10.1186/s12866-023-03045-y (PMC10601334; doi:10.1186/s12866-023-03045-y)
Supplement: Supplementary file 1 — Supplementary Material 1 [file 12866_2023_3045_MOESM1_ESM.docx]

**Materials and Methods**

***Soft coral collection and identification***

The soft coral *Paralemnalia thyrsoides* (Kingdom: Animalia, Phylum: Cnidaria, Class: Anthozoa, Subclass: Octocorallia, Order: Alcyonacea, Family: Nephtheidae) was collected by scuba diving from the coasts of Hurgada, Egypt, at a depth of 12 m, in December 2019 and identified by Prof. Mohamed A. Abu El-Regal, Professor of Biological Oceanography, Marine Biology Department, Faculty of Marine Science, King Abdulaziz University, Jeddah, Saudi Arabia. The collected soft coral was transferred to a sterilized plastic bag containing seawater in an ice container to be transported to the laboratory. Immediately in the laboratory, the collected soft corals were treated for fungal isolation. A voucher specimen was kept in the herbarium of Pharmacognosy Department, Faculty of Pharmacy, Minia University, Minia, Egypt under registration number Mn-Ph-Cog-065.

***Cultivation of pure fungal strains and extraction of fungal cultures***

The pure isolated fugal strains were cultivated on solid rice medium (100 g rice and 120 ml sea water, soaked overnight before being autoclaved) each strain in two of Erlenmeyer flasks (1 L). The fermentation was conducted for 30 days under static conditions at room temperature. Afterwards, extraction of cultures was carried out using ethyl acetate (3 × 500 mL, in each flask) followed by filtration and solvent evaporation under reduced pressure. The obtained crude ethyl acetate extract of each strain was investigated for cytotoxic activity and LC-MS chemical profiling [1].

***Cell viability assay***

A549, CT-26, MDA-MB-231, and U87 cancer cells were plated in 96-well plates at a density of 4X10^4^ cells/well in 100 μL of medium one day prior to adding of different concentrations of the fungal extracts. Untreated control group (n=6) was incubated with 100 µL/well of fresh media and ethyl acetate. After 24 h, the medium was aspirated and replaced by 100 µL of fresh media and 20 µL of MTS reagent in each well (Cell Titer 96 Aqueous One Solution cell proliferation assay, Promega Corporation, Madison, WI, USA), The plates were then incubated at 37 °C with 5% CO_2_ for 2 h. The cells were examined under a cell imaging system (EVOS FL Digital Microscope using a 20X objective. The absorbance was measured at 490 nm using a Spectramax plus 384 Microplate reader (Molecular Devices, Sunnyvale, CA, USA). Relative cell viability values were expressed as the percentage of the absorbance from the treated wells compared to the control wells (untreated), given that the control wells viability was set to 100%. The half maximal inhibitory concentration IC**_50_** (µg/mL) values were obtained using GraphPad Prism 9 software.

***Metabolomics analysis***

Metabolomics profiling of the crude extracts of the fungal cultures was carried out using an Acquity Ultra Performance Liquid Chromatography system coupled to a Synapt G2 HDMS quadrupole time-of-flight hybrid mass spectrometer (Waters, Milford, USA) [2]. Ms converter software was used in order to convert the raw data into divided positive and negative ionization files. Additionally, the obtained files were then subjected to the data mining software MZmine 2.10 (Okinawa Institute of Science and Technology Graduate University, Japan) for deconvolution, peak picking, alignment, deisotoping, and formula prediction. The MarinLit [3] and METLIN [4] databases were finally used for identification of the detected metabolites.

***In silico molecular docking***

Epidermal growth factor receptor tyrosine kinase (EGFR) X-ray crystal structure was downloaded from the Protein Data Bank (PDB ID: 1M17) [5], corrected and 3D protonated at cutoff 15 Å using amber10:EHT force field of Molecular Operating Environment (MOE 2014.0901) software. The binding site was selected at the co-crystallized ligand site at a radius of 4.5 Å then molecular docking was performed using Triangle Matcher, London dG, GBVI/WSA as the placement, rescoring function 1 and 2, respectively as the docking algorithm. The tested compounds were drawn using Chemdraw Ultra 12.0 then transferred as smiles to MOE builder window, added their hydrogens and energy minimized at the same forcefield

| Table S1. Sequence Analysis of the 18S rRNA gene and ITS region by BLASTn Tool | | | | |
| --- | --- | --- | --- | --- |
| Isolates  codes | Name Of Closely Associated Strain | Coverage  % | Identity  % | Gene Bank Accession Number of Closely Associated Strain |
| RD1 | *Penicillium griseofulvum* | 100 | 94.92 | [MF034654.1](https://www.ncbi.nlm.nih.gov/nucleotide/MF034654.1?report=genbank&log$=nucltop&blast_rank=1&RID=3XFDK6FB013) |
| RD2 | *Cladosporium sphaerospermum* | 95 | 100 | [MF374639.1](https://www.ncbi.nlm.nih.gov/nucleotide/MF374639.1?report=genbank&log$=nucltop&blast_rank=2&RID=3XFNAYFH013) |
| RD3 | *Cladosporium liminiforme* | 100 | 98.96 | [MK818536.1](https://www.ncbi.nlm.nih.gov/nucleotide/MK818536.1?report=genbank&log$=nucltop&blast_rank=2&RID=3XFTUEWY01R) |
| RD4 | *Penicillium chrysogenum* | 100 | 98.15 | [MG925208.1](https://www.ncbi.nlm.nih.gov/nucleotide/MG925208.1?report=genbank&log$=nucltop&blast_rank=1&RID=3XG0FEDS01R) |
| RD5 | *Epicoccum nigrum* | 100 | 99.40 | [MN089646.1](https://www.ncbi.nlm.nih.gov/nucleotide/MN089646.1?report=genbank&log$=nucltop&blast_rank=1&RID=3XG9Y2XC013) |

**Table S2. The half-maximal inhibitory concentrations (IC_50_) of fungal extracts for four cancer cell lines:**

| **Cell lines**  **Extracts** | **IC_50_** (µg/mL) | | | | |
| --- | --- | --- | --- | --- | --- |
|  | **RD1** | **RD2** | **RD3** | **RD4** | **RD5** |
| **A549** | 46.73 ±3.24 | 79.20 ±1.16 | 6.99 ± 3.47 | 1.45 ± 8.54 | 91.17 ± 4.3 |
| **CT-26** | 86.84 ±7.67 | 92.28 ±2.64 | 91.17 ± 8 | 1.58 ± 6.55 | 97.01 ± 3. 57 |
| **MDA-MB-231** | 74.65 ±2.87 | 47.56 ±5.53 | 37.50 ±7.73 | 1.39 ± 2.0 | 81.22 ± 1.95 |
| **U87** | 5.507 ±6.79 | 81.41 ±0.568 | 72.73 ±1.596 | 36.28 ±1.49 | 74.27 ± 3.15 |

Table S3: A list of the dereplicated metabolites from the investigated extracts of the five fungal extracts

| No. | RT | M/Z | Compound | Molecular formula | M.wt | Biological source | Ref. |
| --- | --- | --- | --- | --- | --- | --- | --- |
| 1 | 3.891166667 | 243.123 | Penicimonoterpene | **C_12_H_20_O_5_** | 244.131 | *Penicillium chrysogenum* | [6] |
| 2 | 5.769666667 | 329.232 | Penicitide B | **C_18_H_34_O_5_** | 330.240 |  |  |
| 3 | 6.609666667 | 313.237 | Penicitide A | **C_18_H_34_O_4_** | 314.245 |  |  |
| 4 | 3.853 | 259.118 | Penicierythritols B | **C_12_H_20_O_6_** | 260.125 |  | [7] |
| 5 | 6.305 | 637.155 | Chrysoxanthone A | **C_32_H_30_O_14_** | 638.163 |  | [8] |
| 6 | 9.62783333 | 505.351 | Penicisteroid A | **C_30_H_50_O_6_** | 506.360 |  | [9] |
| 7 | 8.4312 | 656.401 | Chrysogeamide E | **C_35_H_55_N_5_O_7_** | 657.410 |  | [10] |
| 8 | 5.672 | 171.102 | Decarestrictine L | **C_9_H_16_O_3_** | 172.109 | *Penicillium simplicissimum* | [11] |
| 9 | 5.308 | 213.148 | Patulolide C | **C_12_H_20_O_3_** | 212.141 | *Penicillium urticae* | [12] |
| 10 | 4.41816666 | 213.112 | Citreoviral | **C_11_H_18_O_4_** | 214.120 | *Penicillium citreoviride* | [13] |
| 11 | 5.36483333 | 309.206 | Cephalosporolide H | **C_18_H_30_O_4_** | 310.214 | *Penicillium sp.* | [14] |
| 12 | 7.80733333 | 323.186 | Hynapene A | **C_18_H_28_O_5_** | 324.193 | *Penicillium sp. FO-1611* | [15] |
| 13 | 5.3255 | 327.216 | Penisporolide A | **C_18_H_30_O_5_** | 326.209 | *Penicillium sp. (HKI Strain No. GT2002605)* | [16] |
| 14 | 5.2924 | 349.200 | Rezishanone B | **C_20_H_28_O_5_** | 348.193 | *Penicillium notatum (GWP A)* | [17] |
| 15 | 7.7254 | 379.158 | Penicitrinone A | **C_23_H_24_O_5_** | 380.162 | *Penicillium citrinum Thom IFM 53298* | [18] |
| 16 | 7.7312 | 381.161 | Penicitrinol A | **C_23_H_26_O_5_** | 382.178 |  |  |
| 17 | 6.305 | 637.155 | Rugulotrosin A | **C_32_H_30_O_14_** | 638.163 | *Penicillium sp.* | [19] |
| 18 |  |  | Rugulotrosin B |  |  |  |  |
| 19 | 7.0148 | 469.261 | Communesin G | **C_29_H_34_N_4_O_2_** | 470.268 | *Penicillium rivulum* | [20] |
| 20 | 8.10716666 | 427.175 | Paraherquonin | **C_24_H_28_O_7_** | 428.183 | *Penicillium paraherquei* | [21] |
| 21 | 7.75875 | 625.396 | Citrinadin A | **C_35_H_52_N_4_O_6_** | 624.388 | *Penicillium citrinum (strain N-059)* | [22] |
| 22 | 8.864666667 | 239.058 | (+)- Epicoccone C | **C_11_H_12_O_6_** | 240.063 | *Epicoccum nigrum* | [23] |
| 23 | 3.906 | 321.124 | (±)-5 hydroxydiphenylalazine A | **C_19_H_18_N_2_O_3_** | 322.131 |  | [24] |
| 24 | 3.465 | 453.025 | amphiepicoccin I | **C_18_H_18_N_2_O_6_S_3_** | 454.0327 |  | [25] |
| 25 | 7.768166667 | 223.026 | Herbaric acid | **C_10_H_8_O_6_** | 224.0320 | *Cladosporium herbarum* | [26] |
| 26 | 4.2515 | 305.150 | Thiocladospolide E | **C_14_H_26_O_5_S** | 306.150 | *Cladosporium spSCNU-F0001* | [27] |
| 27 | 4.004 | 229.144 | Cladoscyclitol C | **C_12_H_22_O_4_** | 230.151 | *Cladosporium spJJM22* | [28] |
| 28 | 4.8498 | 245.138 | Cladoscyclitol D | **C_12_H_22_O_5_** | 246.146 | *Cladosporium sp. JJM22* |  |
| 29 | 5.2285 | 350.202 | Cladosporiumin B | **C_19_H_27_NO_5_** | 349.188 | *Cladosporium sp. SCSIO z0025* | [29] |
| 30 | 6.250166667 | 227.128 | Cladospolide E | **C_12_H_20_O_4_** | 228.136 | *Cladosporium sp. TZP29* | [30] |
| 31 | 6.0846 | 418.208 | (*Z*)-cladosin K | **C_25_H_29_N_3_O_3_** | 419.220 | *Cladosporium sphaerospermum L3P3* | [31] |
| 32 | 3.891166667 | 243.123 | Cladoscyclitol A | **C_12_H_20_O_5_** | 244.131 | *Cladosporium sp. JJM22* | [28] |

**Table S4.** The molecular docking results of the fungi strains isolated **32** compounds using EGFR (using PDB 1M17) against the co-crystallized ligand **AQ4**. The crucial residues were highlighted in bold font.

| **Cpds** | **Binding energy score (kcal/mol)** | **Ligand** | | | **Kinase protein** | | | **Interaction type** | **Distance (Å)** | **Energy (kcal/mol)** |
| --- | --- | --- | --- | --- | --- | --- | --- | --- | --- | --- |
|  |  | **Interacting moieties** | | | **Interacting moieties** | **Amino acid residue** | |  |  |  |
| **AQ4** | -9.52 | C | 19 | | O | **Gln** | **767** | H-donor | 3.15 | -1.0 |
|  |  | N | 2 | | N | **Met** | **769** | H-acceptor | 2.70 | -2.1 |
| **1** | -7.17 | O | 11 | | SD | Met | 742 | H-donor | 4.00 | -0.10 |
|  |  | O | 12 | | OD2 | Asp | 831 | H-donor | 3.58 | -0.20 |
|  |  | O | 10 | | NZ | **Lys** | **721** | H-acceptor | 3.38 | -0.30 |
|  |  | O | 12 | | NZ | **Lys** | **721** | H-acceptor | 3.37 | -1.10 |
| **2** | -9.16 | C | 16 | | SD | Met | 742 | H-donor | 3.62 | -0.70 |
|  |  | C | 17 | | SD | Met | 742 | H-donor | 3.64 | -0.60 |
|  |  | O | 21 | | OE2 | **Glu** | **738** | H-donor | 2.75 | -4.70 |
|  |  | O | 21 | | NZ | **Lys** | **721** | H-acceptor | 3.02 | 0.20 |
| **3** | -8.00 | C | 9 | | SD | Met | 742 | H-donor | 3.81 | -0.50 |
|  |  | O | 14 | | OE2 | **Glu** | **738** | H-donor | 2.88 | -2.50 |
|  |  | O | 14 | | SD | Met | 742 | H-donor | 3.83 | -0.40 |
|  |  | C | 20 | | O | Leu | 694 | H-donor | 3.5 | 0.10 |
|  |  | O | 12 | | NZ | **Lys** | **721** | H-acceptor | 2.98 | -3.80 |
| **4** | -7.63 | C | 3 | | O | **Gln** | **767** | H-donor | 3.51 | -0.50 |
|  |  | O | 6 | | SG | Cys | 751 | H-donor | 3.51 | -1.10 |
|  |  | O | 10 | | SD | Met | 742 | H-donor | 4.19 | -1.20 |
|  |  | O | 12 | | SD | Met | 742 | H-donor | 3.1 | -1.60 |
|  |  | O | 13 | | O | Ala | 719 | H-donor | 3.31 | -0.30 |
|  |  | O | 6 | | CB | Cys | 751 | H-acceptor | 3.31 | -0.30 |
| **5** | -10.33 | C | 28 | | OE1 | Glu | 780 | H-donor | 3.49 | -0.20 |
|  |  | C | 36 | | O | Phe | 771 | H-donor | 3.54 | -0.40 |
|  |  | C | 45 | | O | **Gln** | **767** | H-donor | 3.19 | -0.40 |
|  |  | O | 41 | | OG1 | Thr | 766 | H-acceptor | 2.70 | -0.30 |
|  |  | O | 43 | | OG1 | Thr | 830 | H-acceptor | 2.65 | -2.70 |
|  |  | O | 5 | | NZ | **Lys** | **721** | ionic | 3.22 | -3.20 |
|  |  | 6-ring | | | CB | Leu | 694 | pi-H | 4.39 | -0.40 |
|  |  | 6-ring | | | CD1 | Leu | 694 | pi-H | 4.41 | -0.60 |
| **6** | -9.11 | O | | 30 | SG | Cys | 773 | H-donor | 4.00 | -0.70 |
|  |  | O | | 29 | CB | Arg | 817 | H-acceptor | 3.53 | 0.00 |
|  |  | O | | 35 | CB | **Met** | **769** | H-acceptor | 3.7 | -0.20 |
|  |  | O | | 35 | CA | Gly | 772 | H-acceptor | 3.55 | -0.20 |
|  |  | O | | 31 | 6-ring | Phe | 699 | H-pi | 3.54 | -0.40 |
| **7** | -11.66 | C | | 8 | O | **Met** | **769** | H-donor | 3.48 | -0.40 |
|  |  | N | | 19 | OD2 | Asp | 831 | H-donor | 3.01 | -5.10 |
|  |  | N | | 23 | OD2 | Asp | 831 | H-donor | 3.70 | -0.40 |
|  |  | C | | 24 | O | Arg | 817 | H-donor | 3.28 | -0.50 |
|  |  | C | | 39 | SG | Cys | 773 | H-donor | 3.85 | -0.50 |
|  |  | O | | 10 | N | **Met** | **769** | H-acceptor | 3.12 | -2.10 |
|  |  | O | | 18 | CE | **Lys** | **721** | H-acceptor | 3.07 | -0.60 |
|  |  | C | | 20 | 6-ring | Phe | 699 | H-pi | 4.61 | -0.80 |
|  |  | C | | 43 | 6-ring | Phe | 699 | H-pi | 3.75 | -0.60 |
| **8** | -6.74 | C | | 3 | SD | Met | 742 | H-donor | 3.93 | -0.50 |
|  |  | O | | 12 | OD1 | Asp | 831 | H-donor | 3.40 | -0.30 |
|  |  | O | | 12 | OD2 | Asp | 831 | H-donor | 3.27 | -0.80 |
|  |  | O | | 10 | OG1 | Thr | 830 | H-acceptor | 2.94 | -1.30 |
|  |  | O | | 12 | NZ | **Lys** | **721** | H-acceptor | 3.09 | -4.90 |
| **9** | -6.07 | C | | 8 | OD2 | Asp | 831 | H-donor | 3.40 | 0.80 |
|  |  | O | | 13 | OG1 | Thr | 830 | H-acceptor | 3.17 | -0.70 |
|  |  | O | | 15 | NZ | **Lys** | **721** | H-acceptor | 3.05 | -2.40 |
| **10** | -6.84 | C | | 7 | SG | Cys | 751 | H-donor | 4.09 | -0.30 |
|  |  | O | | 13 | SD | Met | 742 | H-donor | 4.28 | -0.20 |
|  |  | C | | 14 | SD | Met | 742 | H-donor | 3.34 | -0.50 |
|  |  | O | | 15 | OE2 | **Glu** | **738** | H-donor | 2.90 | -4.00 |
|  |  | O | | 6 | OG1 | Thr | 830 | H-acceptor | 2.77 | -1.10 |
|  |  | O | | 13 | OG1 | Thr | 766 | H-acceptor | 2.88 | -1.40 |
|  |  | O | | 15 | NZ | **Lys** | **721** | H-acceptor | 2.81 | -0.30 |
| **11** | -8.1 | C | | 10 | SG | Cys | 751 | H-donor | 3.53 | -0.50 |
|  |  | O | | 16 | OG1 | Thr | 830 | H-acceptor | 2.86 | -1.30 |
| **12** | -6.59 | C | | 9 | OD2 | Asp | 831 | H-donor | 3.40 | -0.70 |
|  |  | O | | 22 | OD1 | Asp | 831 | H-donor | 3.60 | -0.20 |
|  |  | O | | 17 | CA | Gly | 772 | H-acceptor | 3.25 | -0.50 |
|  |  | O | | 18 | N | **Met** | **769** | H-acceptor | 3.40 | -0.50 |
|  |  | O | | 21 | NZ | **Lys** | **721** | H-acceptor | 3.11 | -2.20 |
| **13** | -7.61 | C | | 13 | OD1 | Asp | 831 | H-donor | 3.50 | -0.20 |
|  |  | O | | 19 | SD | Met | 742 | H-donor | 4.44 | -0.10 |
|  |  | O | | 19 | OG1 | Thr | 766 | H-acceptor | 2.79 | -1.80 |
| **14** | -7.61 | C | | 11 | OD1 | Asp | 831 | H-donor | 4.21 | -0.30 |
| **15** | -8.37 | O | | 20 | N | **Met** | **769** | H-acceptor | 3.21 | -0.10 |
|  |  | 6-ring | | | CB | Leu | 694 | pi-H | 4.10 | -0.90 |
|  |  | 6-ring | | | CD1 | Leu | 694 | pi-H | 4.26 | -0.20 |
|  |  | 6-ring | | | CG1 | Val | 702 | pi-H | 4.74 | -0.30 |
|  |  | 6-ring | | | CG1 | Val | 702 | pi-H | 4.13 | -0.70 |
|  |  | 6-ring | | | CA | Gly | 772 | pi-H | 4.68 | -0.30 |
| **16** | -8.21 | O | | 13 | NZ | **Lys** | **721** | H-acceptor | 2.91 | -4.00 |
|  |  | 6-ring | | | CG1 | Val | 702 | pi-H | 4.45 | -0.20 |
|  |  | 6-ring | | | CG2 | Val | 702 | pi-H | 4.21 | -0.60 |
|  |  | 6-ring | | | CD | **Lys** | **721** | pi-H | 4.89 | -0.30 |
|  |  | 6-ring | | | CA | Gly | 772 | pi-H | 4.55 | -0.20 |
| **17** | -10.51 | C | | 12 | OD2 | Asp | 831 | H-donor | 3.63 | -0.20 |
|  |  | O | | 25 | OG1 | Thr | 830 | H-donor | 2.92 | -0.30 |
|  |  | O | | 27 | SG | Cys | 751 | H-donor | 4.26 | -0.20 |
|  |  | C | | 29 | SG | Cys | 751 | H-donor | 3.71 | -1.00 |
|  |  | C | | 29 | OG1 | Thr | 766 | H-donor | 3.25 | -0.10 |
|  |  | O | | 31 | O | **Met** | **769** | H-donor | 3.46 | -0.20 |
|  |  | O | | 24 | CB | Ala | 719 | H-acceptor | 3.02 | -0.40 |
|  |  | O | | 25 | N | Asp | 831 | H-acceptor | 3.28 | -0.10 |
|  |  | 6-ring | | | CD1 | Leu | 694 | pi-H | 3.99 | -0.30 |
|  |  | 6-ring | | | CD1 | Leu | 820 | pi-H | 3.82 | -0.20 |
| **18** | -9.66 | O | | 41 | CD | **Lys** | **721** | H-acceptor | 3.24 | -0.30 |
|  |  | O | | 9 | CA | Asp | 831 | H-acceptor | 3.31 | -0.60 |
|  |  | O | | 45 | NZ | **Lys** | **721** | ionic | 2.67 | -7.10 |
|  |  | O | | 46 | 6-ring | Phe | 699 | H-pi | 3.66 | -0.20 |
|  |  | 6-ring | | | CG1 | Val | 702 | pi-H | 4.22 | -0.50 |
| **19** | -7.88 | C | | 12 | O | Arg | 817 | H-donor | 3.26 | -0.50 |
|  |  | C | | 14 | SG | Cys | 773 | H-donor | 3.35 | -0.40 |
|  |  | C | | 35 | OD1 | Asp | 776 | H-donor | 3.72 | -0.20 |
|  |  | N | | 10 | OD2 | Asp | 831 | ionic | 3.64 | -1.40 |
| **20** | -6.45 | C | | 13 | OD1 | Asp | 831 | H-donor | 2.94 | -0.40 |
|  |  | O | | 30 | OD2 | Asp | 831 | H-donor | 2.56 | -1.20 |
|  |  | O | | 14 | NZ | **Lys** | **721** | H-acceptor | 2.62 | -7.00 |
| **21** | -9.53 | C | | 2 | OD1 | Asp | 776 | H-donor | 3.03 | -0.70 |
|  |  | O | | 31 | O | Leu | 694 | H-donor | 3.03 | -1.10 |
|  |  | C | | 35 | SG | Cys | 773 | H-donor | 3.58 | -0.80 |
|  |  | N | | 34 | OD1 | Asp | 776 | ionic | 3.43 | -2.20 |
|  |  | N | | 43 | OE1 | Glu | 780 | ionic | 3.05 | -4.20 |
| **22** | -6.41 | C | | 4 | OE2 | **Glu** | **738** | H-donor | 3.19 | -0.50 |
|  |  | C | | 4 | SD | Met | 742 | H-donor | 3.43 | -1.00 |
|  |  | O | | 12 | OD2 | Asp | 831 | H-donor | 3.20 | -1.80 |
|  |  | 6-ring | | | CD | **Lys** | **721** | pi-H | 3.94 | -0.20 |
|  |  | 6-ring | | | NZ | **Lys** | **721** | pi-cation | 4.68 | -0.30 |
| **23** | -8.29 | 6-ring | | | CD | **Lys** | **721** | pi-H | 3.77 | -0.20 |
|  |  | 6-ring | | | OG1 | Thr | 766 | pi-H | 3.63 | -0.20 |
| **24** | -7.91 | O | | 13 | SG | Cys | 751 | H-donor | 3.77 | -0.80 |
|  |  | O | | 45 | OD2 | Asp | 831 | H-donor | 2.91 | -1.00 |
|  |  | S | | 28 | CG2 | Val | 702 | H-acceptor | 3.99 | -0.50 |
|  |  | S | | 44 | CB | **Lys** | **721** | H-acceptor | 4.29 | -0.30 |
|  |  | O | | 45 | N | Asp | 831 | H-acceptor | 3.22 | 0.10 |
| **25** | -6.96 | O | | 15 | OE2 | **Glu** | **738** | H-donor | 3.07 | -2.40 |
|  |  | O | | 15 | SD | Met | 742 | H-donor | 4.18 | -0.40 |
|  |  | O | | 13 | CA | Leu | 768 | H-acceptor | 3.71 | -0.20 |
|  |  | O | | 13 | N | **Met** | **769** | H-acceptor | 3.03 | -4.90 |
| **26** | -8.26 | C | | 16 | OD2 | Asp | 831 | H-donor | 3.45 | -0.50 |
|  |  | O | | 18 | OD1 | Asp | 831 | H-donor | 2.90 | -3.00 |
|  |  | O | | 20 | SG | Cys | 751 | H-donor | 4.40 | -0.20 |
|  |  | O | | 20 | O | Gln | 767 | H-donor | 3.46 | -0.40 |
|  |  | S | | 15 | CD | **Lys** | **721** | H-acceptor | 3.74 | -0.50 |
| **27** | -7.35 | C | | 2 | SD | Met | 742 | H-donor | 3.76 | -0.40 |
|  |  | C | | 7 | OD2 | Asp | 831 | H-donor | 3.60 | -0.30 |
|  |  | O | | 14 | OD2 | Asp | 831 | H-donor | 2.66 | -1.20 |
|  |  | O | | 15 | OE2 | **Glu** | **738** | H-donor | 2.77 | -3.90 |
|  |  | O | | 8 | NZ | **Lys** | **721** | H-acceptor | 3.44 | -0.10 |
|  |  | O | | 14 | N | Asp | 831 | H-acceptor | 3.04 | -0.50 |
|  |  | O | | 16 | OG1 | Thr | 766 | H-acceptor | 3.06 | -0.30 |
| **28** | -7.99 | O | | 8 | OD2 | Asp | 831 | H-donor | 3.08 | -0.50 |
|  |  | O | | 14 | SD | Met | 742 | H-donor | 3.37 | -1.60 |
|  |  | O | | 15 | SD | Met | 742 | H-donor | 3.07 | -1.80 |
|  |  | O | | 8 | NZ | **Lys** | **721** | H-acceptor | 3.07 | -5.20 |
|  |  | O | | 17 | OG1 | Thr | 766 | H-acceptor | 2.89 | -0.60 |
| **29** | -8.87 | O | | 7 | CA | Leu | 768 | H-acceptor | 3.33 | -0.40 |
|  |  | O | | 7 | N | **Met** | **769** | H-acceptor | 2.80 | -3.00 |
|  |  | O | | 18 | OG1 | Thr | 830 | H-acceptor | 2.79 | -0.40 |
|  |  | C | | 24 | 6-ring | Phe | 699 | H-pi | 3.61 | -0.40 |
| **30** | -6.20 | C | | 6 | OE2 | **Glu** | **738** | H-donor | 3.25 | -0.90 |
|  |  | O | | 4 | NZ | **Lys** | **721** | H-acceptor | 3.17 | -4.80 |
|  |  | O | | 13 | OG1 | Thr | 766 | H-acceptor | 3.08 | -0.40 |
| **31** | -9.79 | O | | 24 | N | **Met** | **769** | H-acceptor | 3.27 | -1.40 |
|  |  | 5-ring | | | CD | **Lys** | **721** | pi-H | 4.24 | -0.80 |
|  |  | 6-ring | | | N | Cys | 773 | pi-H | 4.30 | -1.00 |
| **32** | -7.79 | O | | 8 | OD2 | Asp | 831 | H-donor | 3.04 | -0.60 |
|  |  | O | | 14 | SD | Met | 742 | H-donor | 3.33 | -0.90 |
|  |  | O | | 16 | SD | Met | 742 | H-donor | 3.08 | -1.90 |
|  |  | O | | 8 | NZ | **Lys** | **721** | H-acceptor | 3.03 | -5.60 |
|  |  | O | | 15 | OG1 | Thr | 766 | H-acceptor | 2.86 | -0.70 |


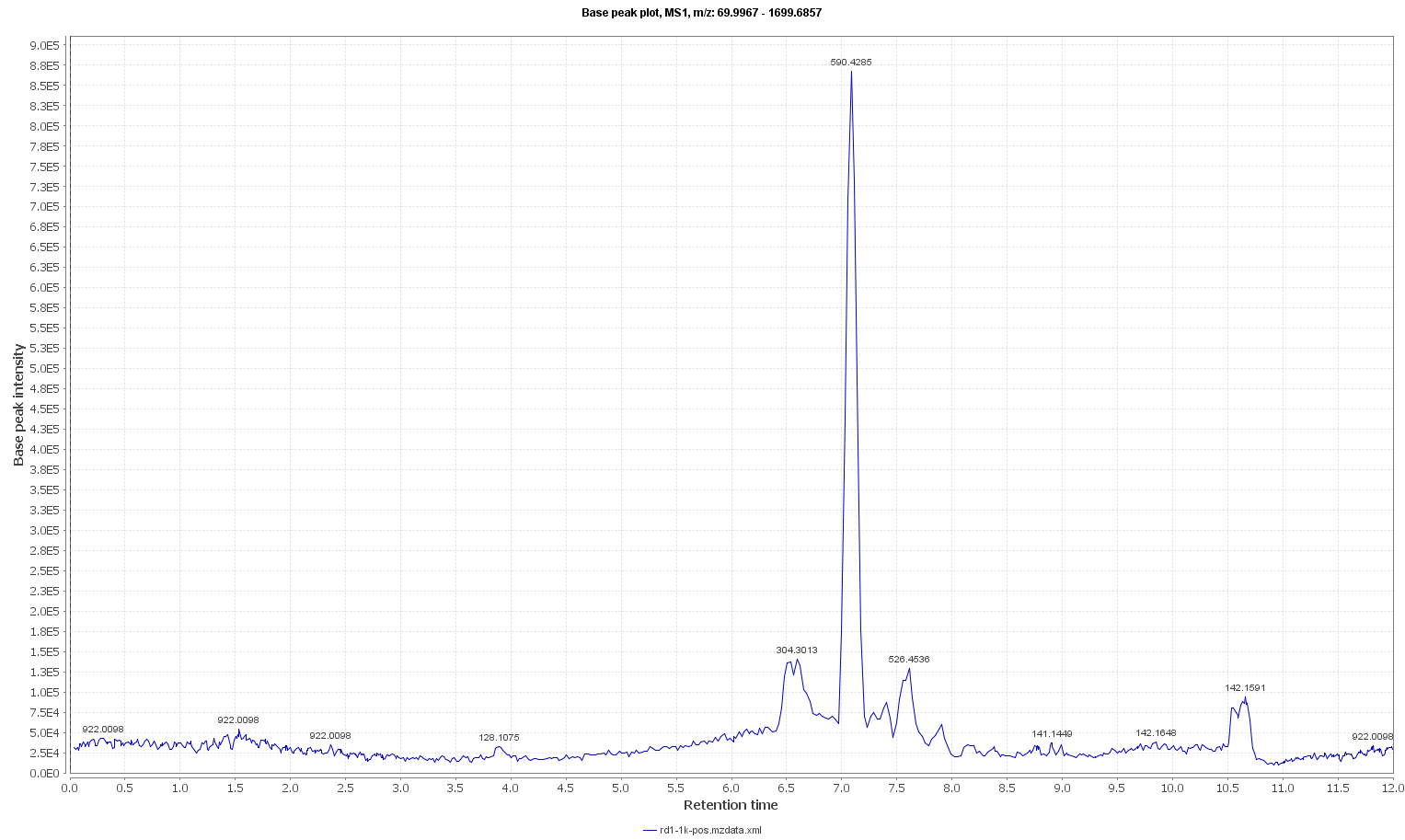


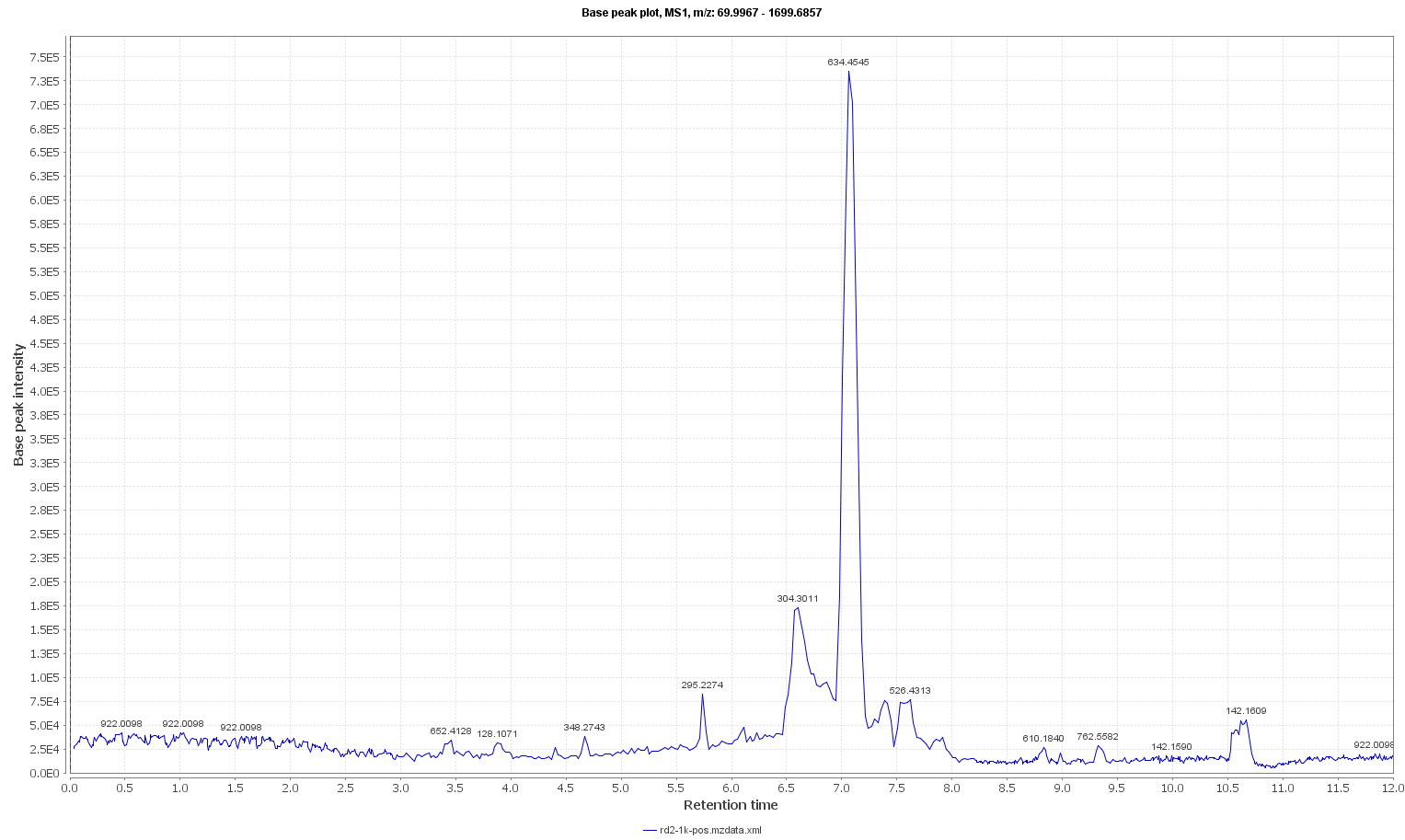


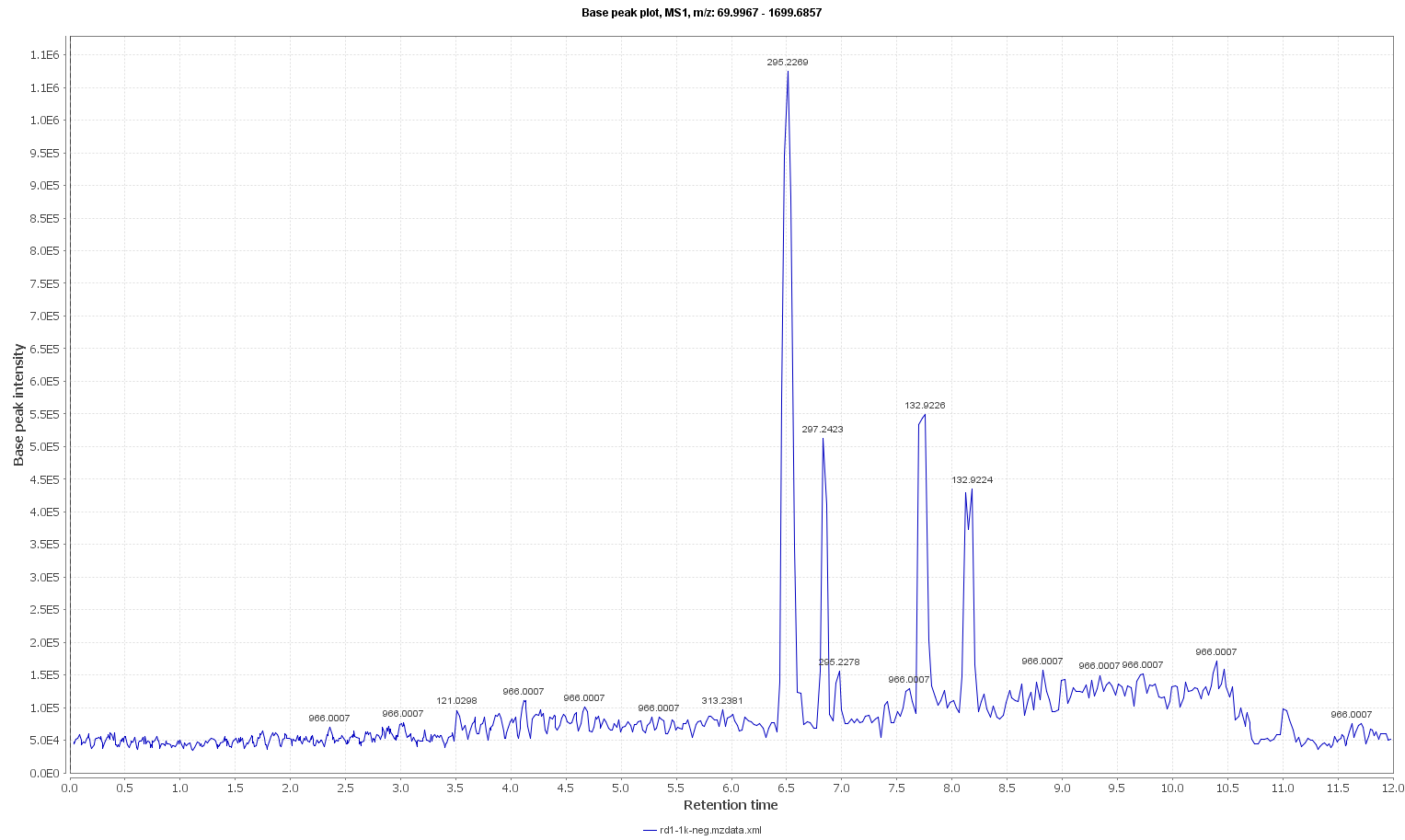


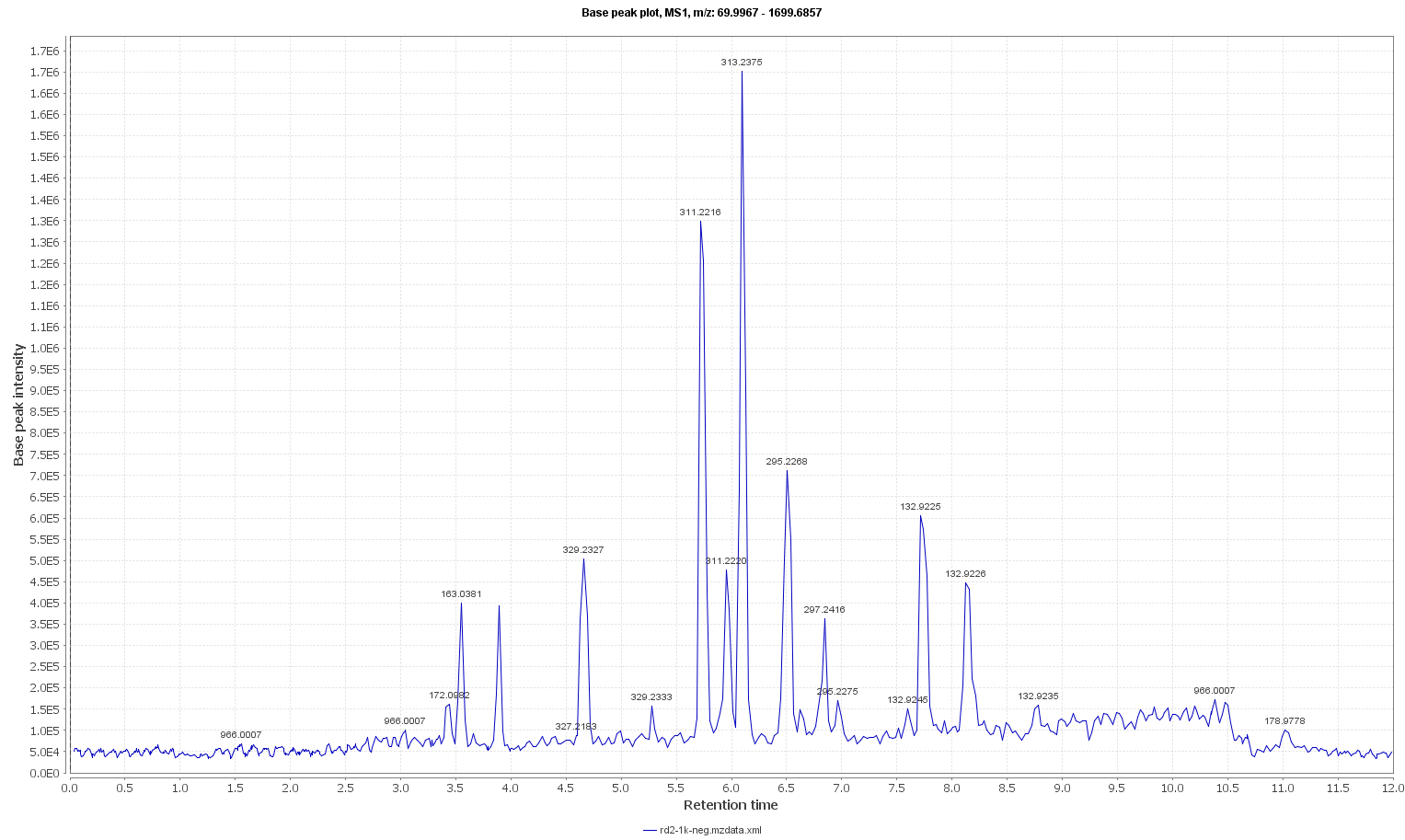


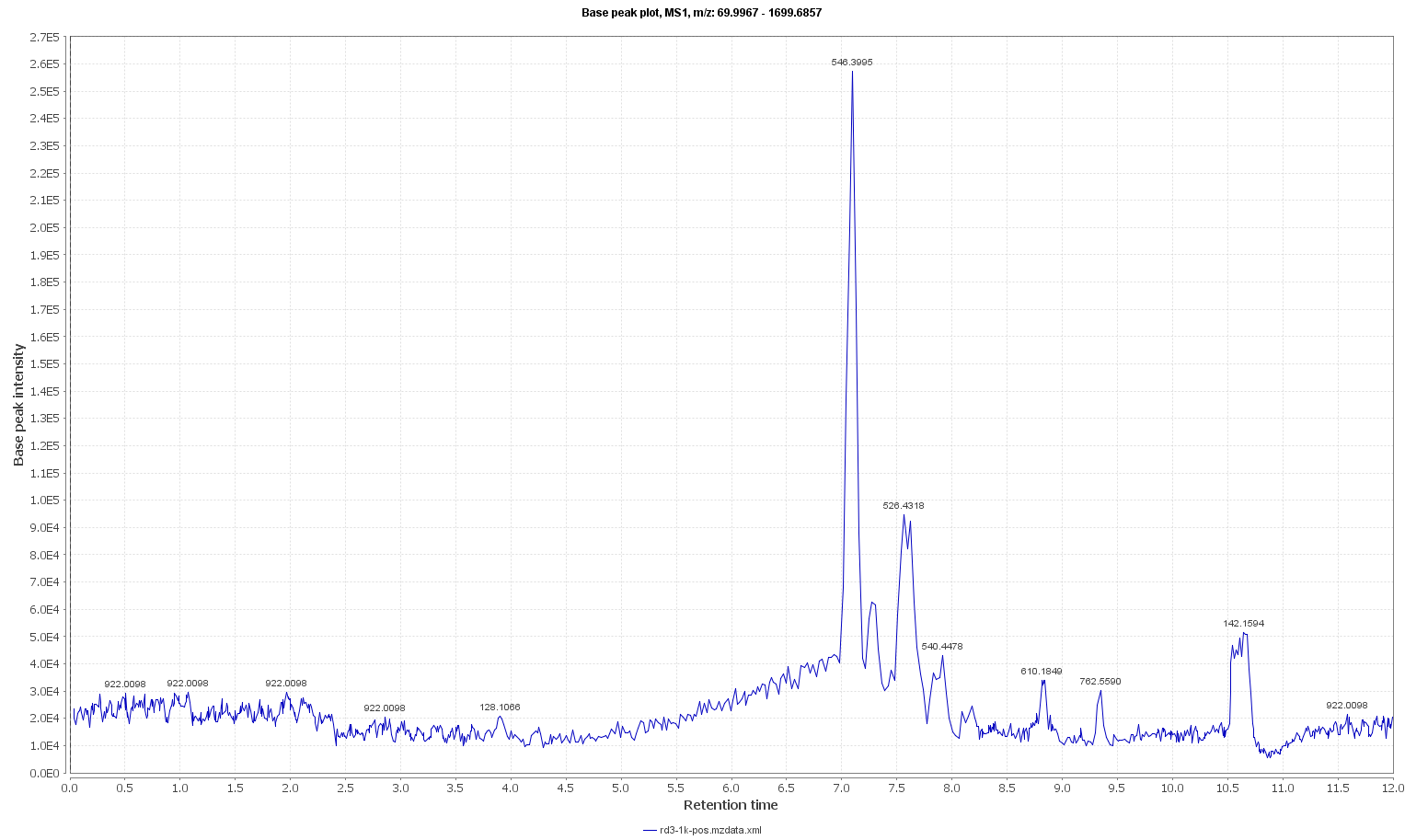


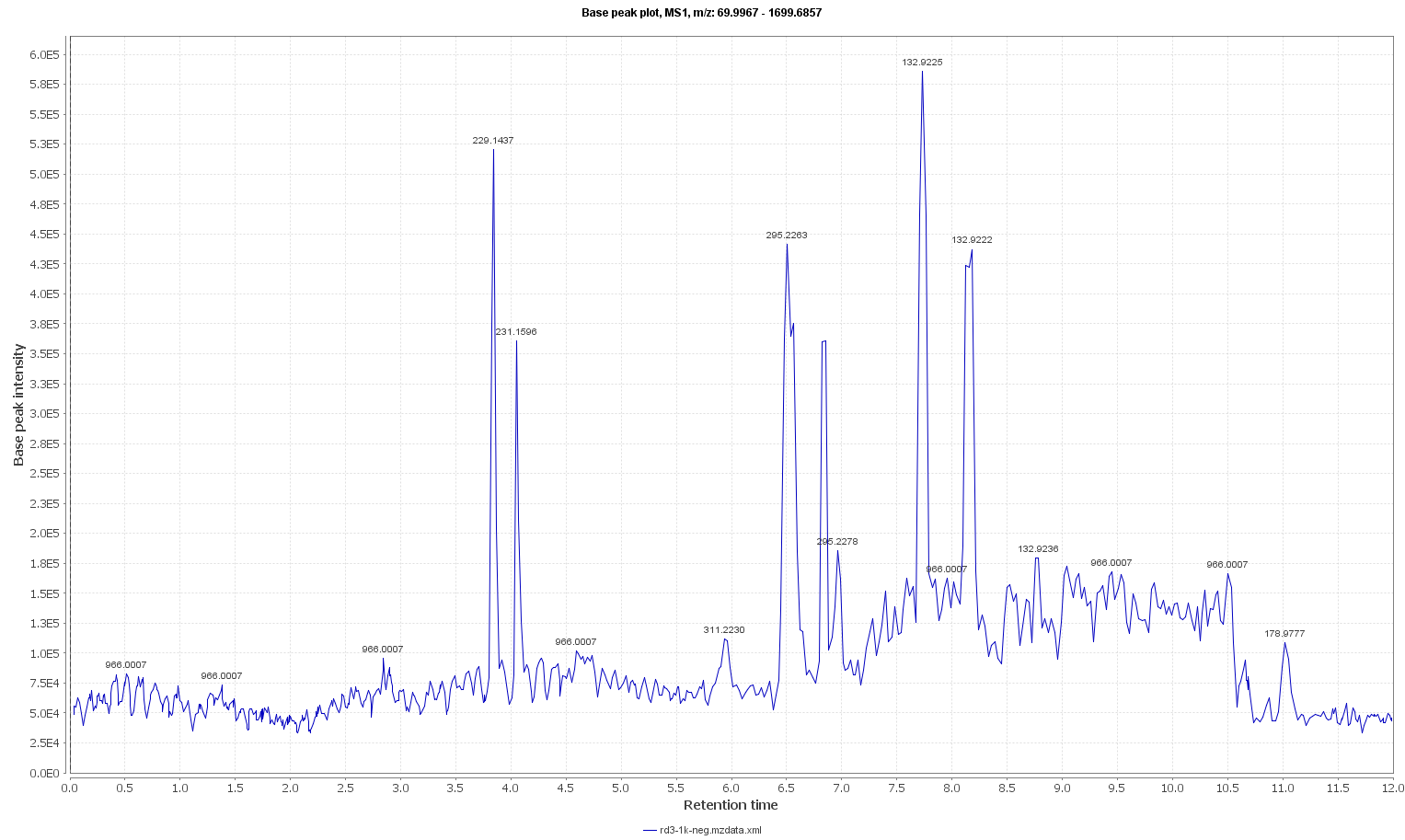


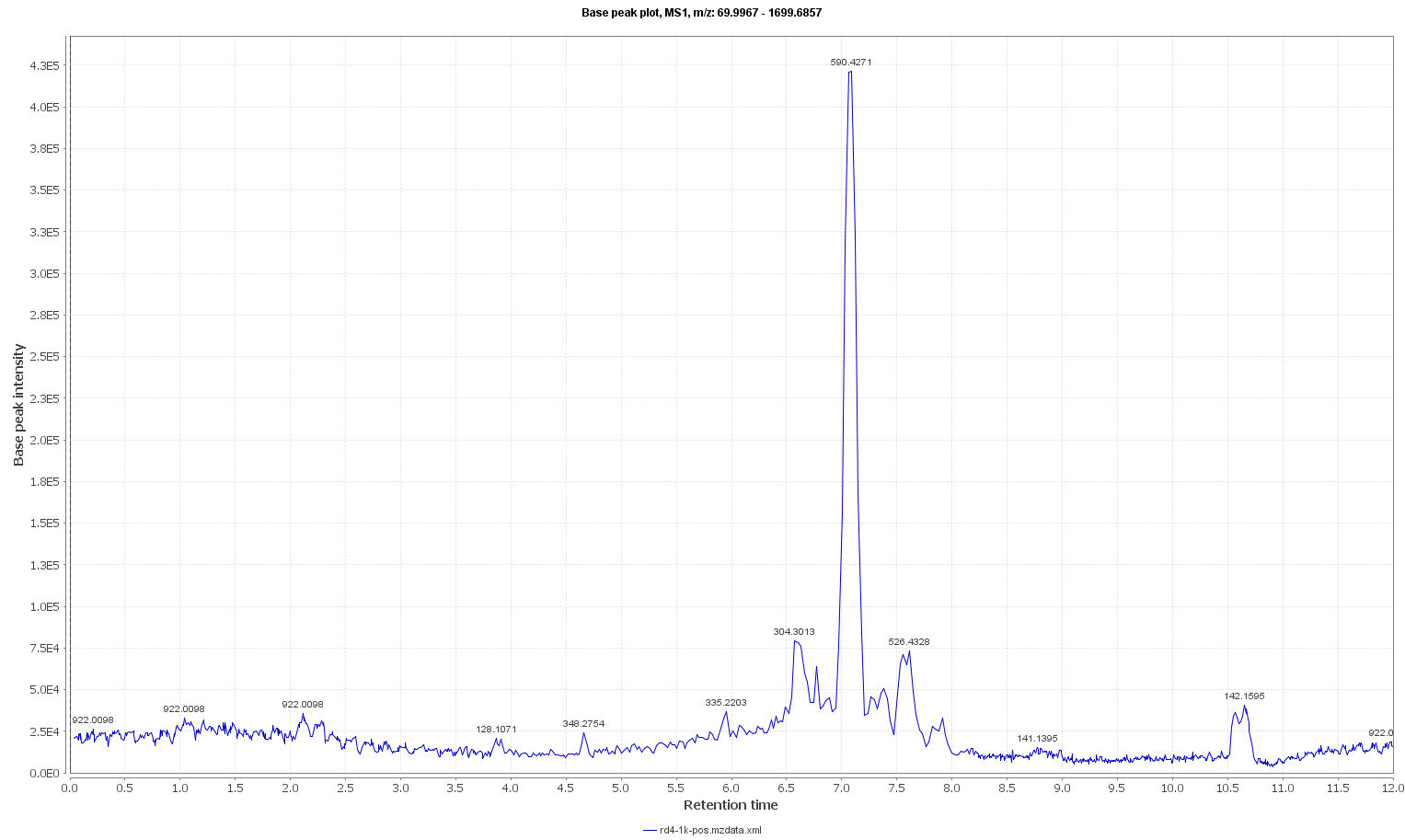


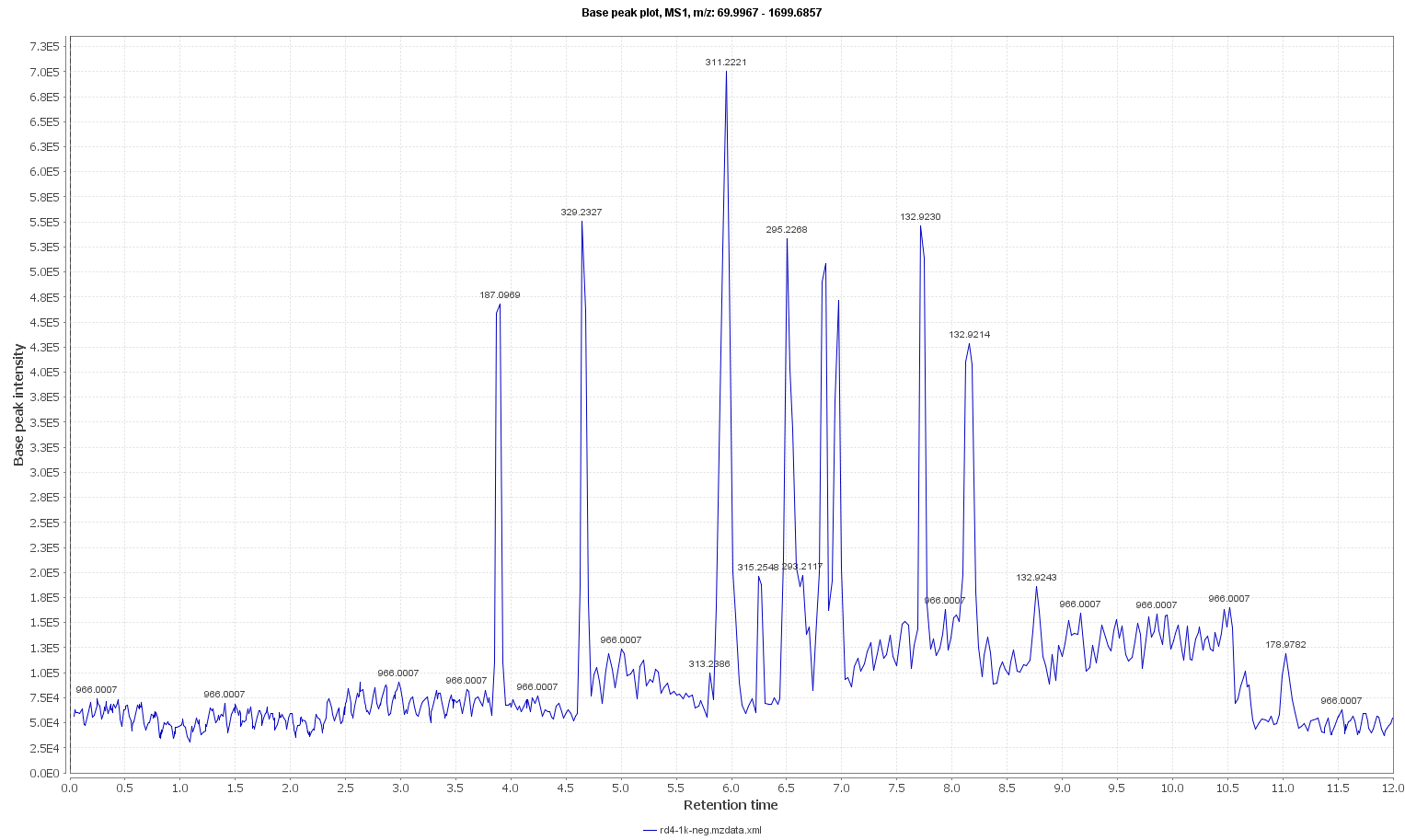


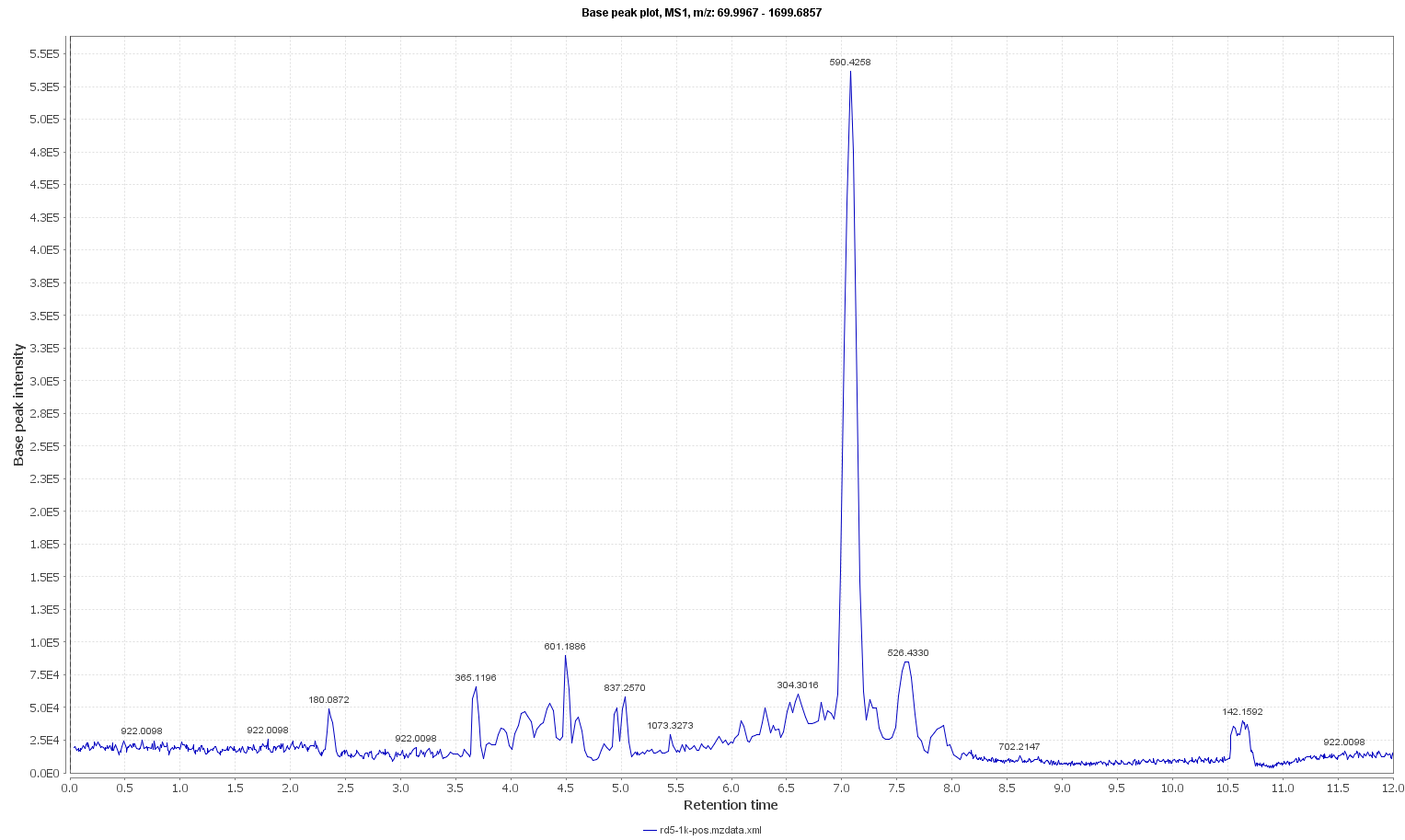


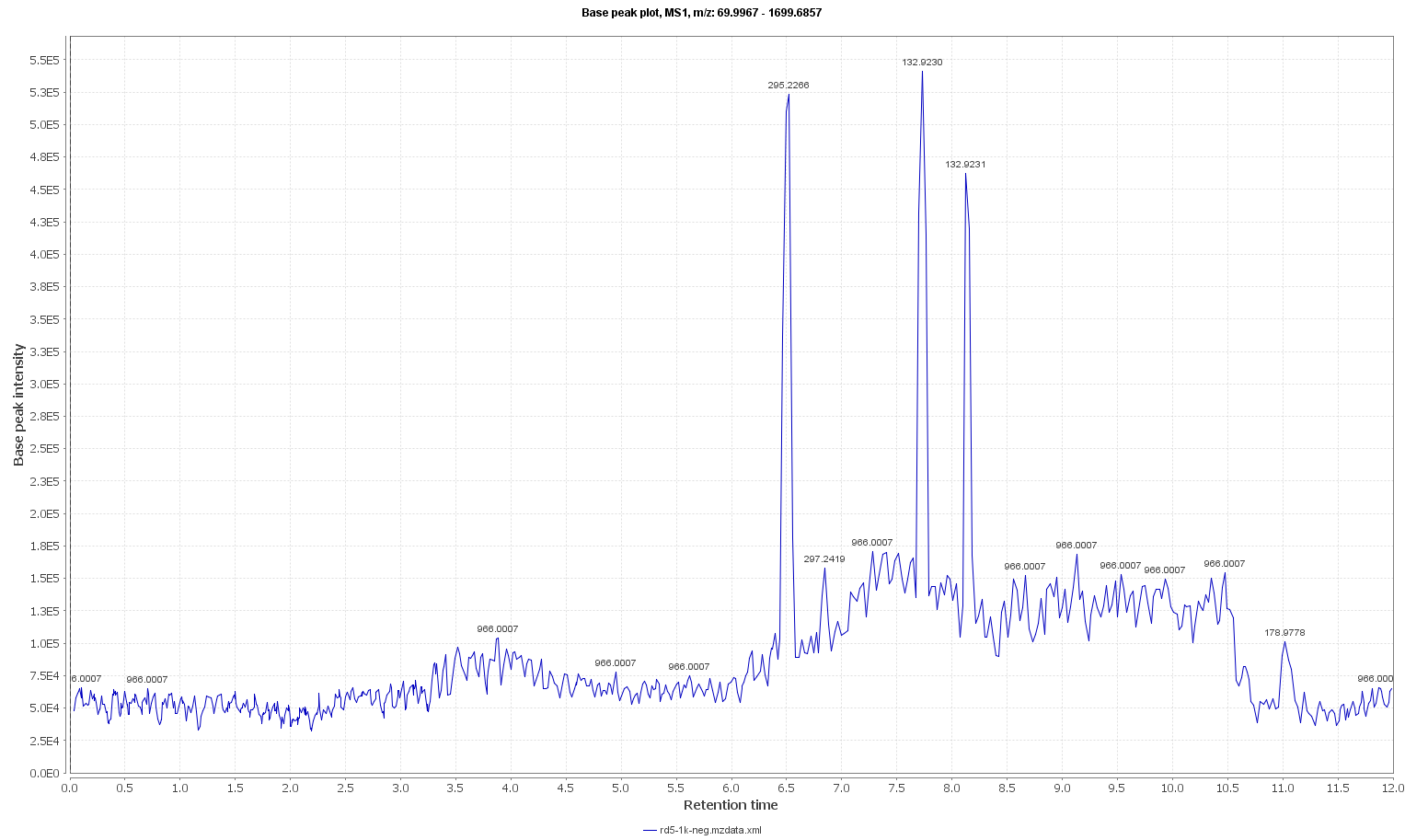


**Figure S1: Total ion chromatograms of the ethyl acetate extracts of the fungal strains.**

**References**

1. Abdelgawad MA, Hamed AA, Nayl AA, Badawy MSE, Ghoneim MM, Sayed AM, et al. The Chemical Profiling, Docking Study, and Antimicrobial and Antibiofilm Activities of the Endophytic fungi Aspergillus sp. AP5. Molecules. 2022;27(5):1704.

2. Abdelmohsen UR, Cheng C, Viegelmann C, Zhang T, Grkovic T, Ahmed S, et al. Dereplication strategies for targeted isolation of new antitrypanosomal actinosporins A and B from a marine sponge associated-Actinokineospora sp. EG49. Marine drugs. 2014;12(3):1220-44.

3. <http://pubs.rsc.org/MarinLit/>. Septemper 2021.

4. <http://metlin.scripps.edu/index.php>. Septemper 2021.

5. Stamos J, Sliwkowski MX, Eigenbrot C. Structure of the epidermal growth factor receptor kinase domain alone and in complex with a 4-anilinoquinazoline inhibitor. Journal of biological chemistry. 2002;277(48):46265-72.

6. Gao S-S, Li X-M, Du F-Y, Li C-S, Proksch P, Wang B-G. Secondary metabolites from a marine-derived endophytic fungus Penicillium chrysogenum QEN-24S. Marine drugs. 2010;9(1):59-70.

7. Xu K, Wei X-L, Xue L, Zhang Z-F, Zhang P. Antimicrobial meroterpenoids and erythritol derivatives isolated from the marine-algal-derived endophytic fungus Penicillium chrysogenum XNM-12. Marine drugs. 2020;18(11):578.

8. Zhen X, Gong T, Wen Y-H, Yan D-J, Chen J-J, Zhu P. Chrysoxanthones A–C, three new Xanthone–Chromanone heterdimers from sponge-associated Penicillium chrysogenum HLS111 treated with histone deacetylase inhibitor. Marine drugs. 2018;16(10):357.

9. Gao S-S, Li X-M, Li C-S, Proksch P, Wang B-G. Penicisteroids A and B, antifungal and cytotoxic polyoxygenated steroids from the marine alga-derived endophytic fungus Penicillium chrysogenum QEN-24S. Bioorganic & medicinal chemistry letters. 2011;21(10):2894-7.

10. Hou X-M, Li Y-Y, Shi Y-W, Fang Y-W, Chao R, Gu Y-C, et al. Integrating molecular networking and 1H NMR to target the isolation of chrysogeamides from a library of marine-derived Penicillium fungi. The Journal of Organic Chemistry. 2019;84(3):1228-37.

11. GRABLEY S, HAMMANN P, HÜTTER K, KIRSCH R, KLUGE H, THIERICKE R, et al. SECONDARY METABOLITES BY CHEMICAL SCREENING. 20 DECARESTRICTINES, A NEW FAMILY OF INHIBITORS OF CHOLESTEROL BIOSYNTHESIS FROM PENICILLIUM: III. DECARESTRICTINES E TO M. The Journal of Antibiotics. 1992;45(7):1176-81.

12. RODPHAYA D, SEKIGUCHI J, YAMADA Y. New macrolides from Penicillium urticae mutant S11R59. The Journal of Antibiotics. 1986;39(5):629-35.

13. Shizuri Y, Nishiyama S, Imai D, Yamamura S, Furukawa H, Kawai K, et al. Isolation and stereostructures of citreoviral, citreodiol, and epicitreodiol. Tetrahedron letters. 1984;25(42):4771-4.

14. Li X, Yao Y, Zheng Y, Sattler I, Lin W. Cephalosporolides H and I, two novel lactones from a marine-derived fungus, penicillium sp. Archives of pharmacal research. 2007;30(7):812-5.

15. Tabata N, Tomoda H, Masuma R, Haneda K, Iwai Y, Omura S. Hynapenes A, B and C, new anticoccidial agents produced by Penicillium sp. I. Production, isolation and physico-chemical and biological properties. The Journal of Antibiotics. 1993;46(12):1849-53.

16. Li X, Sattler I, Lin W. Penisporolides A and B, two new spiral lactones from the marine-derived fungus Penicillium sp. The Journal of Antibiotics. 2007;60(3):191-5.

17. Maskey RP, Grün-Wollny I, Laatsch H. Sorbicillin analogues and related dimeric compounds from Penicillium n otatum. Journal of natural products. 2005;68(6):865-70.

18. Wakana D, Hosoe T, Itabashi T, Okada K, de Campos Takaki GM, Yaguchi T, et al. New citrinin derivatives isolated from Penicillium citrinum. Journal of Natural Medicines. 2006;60(4):279-84.

19. Stewart M, Capon RJ, White JM, Lacey E, Tennant S, Gill JH, et al. Rugulotrosins A and B: Two new antibacterial metabolites from an Australian isolate of a Penicillium sp. Journal of natural products. 2004;67(4):728-30.

20. Dalsgaard PW, Blunt JW, Munro MH, Frisvad JC, Christophersen C. Communesins G and H, New Alkaloids from the Psychrotolerant Fungus Penicillium r ivulum. Journal of natural products. 2005;68(2):258-61.

21. Okuyama E, Yamazaki M, Kobayashi K, Sakurai T. Paraherquonin, a new meroterpenoid from Penicillium paraherquei. Tetrahedron Letters. 1983;24(30):3113-4.

22. Tsuda M, Kasai Y, Komatsu K, Sone T, Tanaka M, Mikami Y, et al. Citrinadin A, a Novel Pentacyclic Alkaloid from Marine-Derived Fungus Penicillium c itrinum. Organic Letters. 2004;6(18):3087-9.

23. Yan Z, Huang C, Guo H, Zheng S, He J, Lin J, et al. Isobenzofuranone monomer and dimer derivatives from the mangrove endophytic fungus Epicoccum nigrum SCNU-F0002 possess α-glucosidase inhibitory and antioxidant activity. Bioorganic Chemistry. 2020;94:103407.

24. Chi L-P, Li X-M, Li L, Li X, Wang B-G. Cytotoxic thiodiketopiperazine derivatives from the deep sea-derived fungus Epicoccum nigrum SD-388. Marine drugs. 2020;18(3):160.

25. Wang Q, Zhang K, Wang W, Zhang G, Zhu T, Che Q, et al. Amphiepicoccins A–J: epipolythiodioxopiperazines from the fish-gill-derived fungus Epicoccum nigrum HDN17-88. Journal of natural products. 2020;83(2):524-31.

26. Jadulco R, Brauers G, Edrada RA, Ebel R, Wray V, Sudarsono a, et al. New metabolites from sponge-derived fungi curvularia l unata and cladosporium h erbarum. Journal of natural products. 2002;65(5):730-3.

27. Huang C, Chen T, Yan Z, Guo H, Hou X, Jiang L, et al. Thiocladospolide E and cladospamide A, novel 12-membered macrolide and macrolide lactam from mangrove endophytic fungus Cladosporium sp. SCNU-F0001. Fitoterapia. 2019;137:104246.

28. Zhang B, Wu J-T, Zheng C-J, Zhou X-M, Yu Z-X, Li W-S, et al. Bioactive cyclohexene derivatives from a mangrove-derived fungus Cladosporium sp. JJM22. Fitoterapia. 2021;149:104823.

29. Huang Z-h, Nong X-h, Liang X, Qi S-h. New tetramic acid derivatives from the deep-sea-derived fungus Cladosporium sp. SCSIO z0025. Tetrahedron. 2018;74(21):2620-6.

30. Zhu M, Gao H, Wu C, Zhu T, Che Q, Gu Q, et al. Lipid-lowering polyketides from a soft coral-derived fungus Cladosporium sp. TZP29. Bioorganic & Medicinal Chemistry Letters. 2015;25(17):3606-9.

31. Zhang Z, He X, Wu G, Liu C, Lu C, Gu Q, et al. Aniline-tetramic acids from the deep-sea-derived fungus Cladosporium sphaerospermum L3P3 cultured with the HDAC inhibitor SAHA. Journal of natural products. 2018;81(7):1651-7.
